# MASH Background Confers Enhanced Disease Susceptibility and Acetaminophen Toxicity in iPSC‐Derived Liver Organoids

**DOI:** 10.1002/advs.202521373

**Published:** 2026-07-27

**Authors:** Ekta Minocha, Ashwani Kumar Gupta, Nate Schmidt, Richard M. Green, John G. Purdy, Jason A. Wertheim

**Affiliations:** ^1^ Department of Surgery College of Medicine University of Arizona Tucson Arizona USA; ^2^ Bio5 Institute University of Arizona Tucson Arizona USA; ^3^ Surgery Service Southern Arizona VA Health Care System Tucson Arizona USA; ^4^ Department of Immunobiology University of Arizona College of Medicine Tucson Arizona USA; ^5^ Northwestern University Feinberg School of Medicine Chicago Illinois USA; ^6^ Department of Biomedical Engineering University of Arizona Tucson Arizona USA

**Keywords:** air liquid interface, differentiation, disease modeling, drug toxicity, engraftment, lipidomics, liver, organoid, pluripotent stem cells, vasculature

## Abstract

Organoids derived from human induced pluripotent stem cells (iPSCs) serve as advanced multicellular models for studying human organ development and disease. Recent liver organoid platforms focus on achieving multicellular organization while minimizing reliance on xenogeneic extracellular matrices to support future clinical translation. Building on these advances, this study establishes a xenogeneic‐free strategy that develops the hepatic cellular repertoire with interdigitating vasculature using an air‐liquid interface approach, generating highly vascularized, multicellular, and functional liver organoids from de‐identified control and metabolic dysfunction‐associated steatohepatitis (MASH) donor‐derived iPSCs. Phenotypic and functional characterization confirms the presence of hepatocytes, cholangiocytes, stellate cells, sinusoidal endothelial cells, and Kupffer‐like cells. These organoids demonstrate the capacity to model steatohepatitis following free‐fatty acid exposure and predict acetaminophen‐induced drug toxicity. Organoids derived from MASH‐donors exhibit increased susceptibility to steatosis, inflammation, fibrosis, and acetaminophen‐induced toxicity. Lipidomic profiling reveals that MASH phenotype in organoids induces global lipidomic shifts that closely resemble those observed in MASH liver biopsies. Further post‐transplantation into mice, the organoids retain hepatic cell repertoire, establish functional anastomoses with host vasculature, display intraluminal erythrocytes, and secrete human‐specific albumin, validating their translational potential. This approach provides a robust, xenogeneic‐free platform for disease modeling, evaluating drug responses, and exploring regenerative therapies.

## Graphical abstract

This work establishes a novel method for generating multicellular liver organoids from control and MASH donor iPSCs. The model recapitulates several disease‐specific characteristics, with MASH donor‐derived organoids showing higher susceptibility. Lipidomic profiling of MASH organoids closely resembles MASH liver biopsies. The organoids demonstrate transplantation potential with well‐preserved cellular identities. This approach eliminates reliance on Matrigel embedding or genetic manipulation.

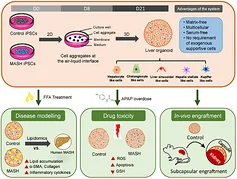

**Abbreviations**
ALI: air‐liquid interface
APA: Pacetaminophen
CDFDA: 5‐(and‐6) carboxy 2’;7’dichlorofluoroscein diacetate
ECM: extracellular matrix
FFA: free‐fatty acid
iPSC: induced pluripotent stem cells
LC/MS‐MS: liquid chromatography high‐resolution tandem mass spectrometry
LPS: lipopolysaccharide
LSECs: liver sinusoidal endothelial‐like cells
MASH: metabolic dysfunction‐associated steatohepatitis
NSG: NOD/SCID/γ‐chain knockout
OA: oleic acid
TUNEL: Terminal Deoxynucleotidyl Transferase‐mediated dUTP Nick End Labeling
UEA: IUlex Europaeus Agglutinin I

## 1 Introduction

Liver disease accounts for approximately two million deaths worldwide each year, highlighting a significant healthcare burden that remains sub‐optimally managed due to limited availability of donor organs and incomplete understanding of disease progression [1]. The lack of appropriate hepatic models that recapitulate human disease phenotypes have been the major obstacles that hinder progress in liver disease research. Traditional animal models, while widely used, cannot fully capture the complexities of human disease pathogenesis and drug response due to differences in their physiology, immune response, and genetic makeup [2]. Likewise, primary human hepatocytes degenerate in *ex vivo* culture, and hepatocyte‐like cells differentiated from pluripotent stem cells lack the necessary cellular interactions with secondary cells of the liver that are needed for accurate disease modeling. To address these limitations, human organoid models composed of multiple liver‐specific cell types offer a promising alternative by circumventing species‐specific differences associated with animal models [3]. Moreover, the possibility of deriving organoid models from patient‐derived cells opens new opportunities for the study of liver diseases as they retain the genetic background of the original patient.

Liver organoids have been generated from human induced pluripotent stem cells (iPSCs) or adult liver stem cells [4, 5, 6, 7, 8]. However, organoids generated in these studies either fail to incorporate all liver cell types or involve mixing of exogenous cells isolated from multiple postnatal donors that may confer an increased risk of rejection or sensitization against host antigens [5, 8, 9, 10]. Moreover, most liver organoid protocols involve embedding cell aggregates in animal‐derived matrices, such as Matrigel [6, 10, 11, 12, 13], which has a poorly defined composition with batch‐to‐batch variability, making it unsuitable for downstream transplant applications. Thus, exploring alternatives to generate multilineage vascularized liver organoids in a Matrigel‐free environment remains a key area of focus. Although some efforts have been made to generate matrigel‐free liver organoids using rotating wall vessels or orbital shakers, a major obstacle is the inability to precisely regulate organoid size, as the cells self‐organize into spheroids of variable sizes within suspension culture [14, 15].

In this study, we describe a novel method to generate highly vascularized, multicellular, functional liver organoids from iPSCs using an air‐liquid interface (ALI) approach that led to a liver‐like cellular repertoire. This strategy also eliminates the need for Matrigel embedding. We report a stepwise strategy for simultaneous induction of endoderm and mesoderm, which results in concomitant differentiation of endoderm‐derived parenchymal cell populations and mesoderm‐derived non‐parenchymal cells, thus obviating the need for exogenous cell introduction or genetic manipulation. These multicellular liver organoids were efficiently generated from iPSCs derived from normal and metabolic dysfunction‐associated steatohepatitis (MASH) backgrounds. To highlight the clinical relevance of our liver organoid model, we investigated its utility in disease modeling and drug‐toxicity. We further conducted lipidomic profiling of control and MASH donor‐derived organoids, comparing their lipid compositions with the lipidomic results of liver biopsies from control and MASH subjects. The MASH organoids exhibited global lipidomic alterations that closely mirrored the profiles observed in MASH patient liver tissues, reinforcing the disease relevance of our model. To evaluate the translational potential, we engrafted liver organoids under the kidney capsule of NOD/SCID/γ‐chain knockout (NSG) mice. All hepatic cell types were preserved at four weeks post‐transplant and were accompanied by the detection of human albumin in the mouse bloodstream along with the presence of mouse erythrocytes within human‐derived vascular lumens, further validating the successful engraftment.

## 2 Results

### 2.1 Generation of Liver Organoids From Human Induced Pluripotent Stem Cells

Liver organoids were generated from human iPSCs derived from donors with MASH and normal controls, using a cocktail of growth factors in a developmentally relevant sequence to mimic in vivo liver development (Figure 1A). Sequential morphological changes during iPSC differentiation into liver organoids are illustrated in Figure 1B. We examined the pluripotent stage of human iPSCs before initiating differentiation at day 0 by immunofluorescence and found that cells uniformly co‐expressed pluripotency markers: OCT4 and TRA‐1‐60 (Figure 1C and Figure S1A for each individual iPSC line). To initiate developmental programing, iPSCs were subjected to WNT activation using GSK3 inhibitor CHIR99021, which promoted an exit from pluripotency to the primitive streak (PS) stage. These cells were responsive to BMP4, resulting in mesodermal cell‐derivatives, and Activin A, resulting in definitive endoderm cells. Immunofluorescence analysis on day 4 confirmed the presence of endodermal (GATA4 and FOXA2) and mesodermal (TBXT) markers, with only a few cells expressing ectoderm marker (MAP2) [16] (Figure 1D and Figure S1B), indicating successful concomitant differentiation into definitive endoderm and mesoderm. qPCR results further supported this observation, showing robust upregulation of endoderm‐associated genes (*GATA4, SOX17*, and *FOXA2*), and mesoderm‐associated genes (*EOMES* and *MIXL1*), along with downregulation of ectodermal (*SOX1* and *PAX6*) and pluripotency (*OCT4* and *NANOG*) markers by day 4 (Figure 1E) [8, 17, 18]. By day 8, the expression of early endodermal and mesodermal markers had declined, while hepatic markers like HNF4α and AFP increased, reflecting progression toward hepatic lineage commitment [19] (Figure 1E).

**FIGURE 1:**
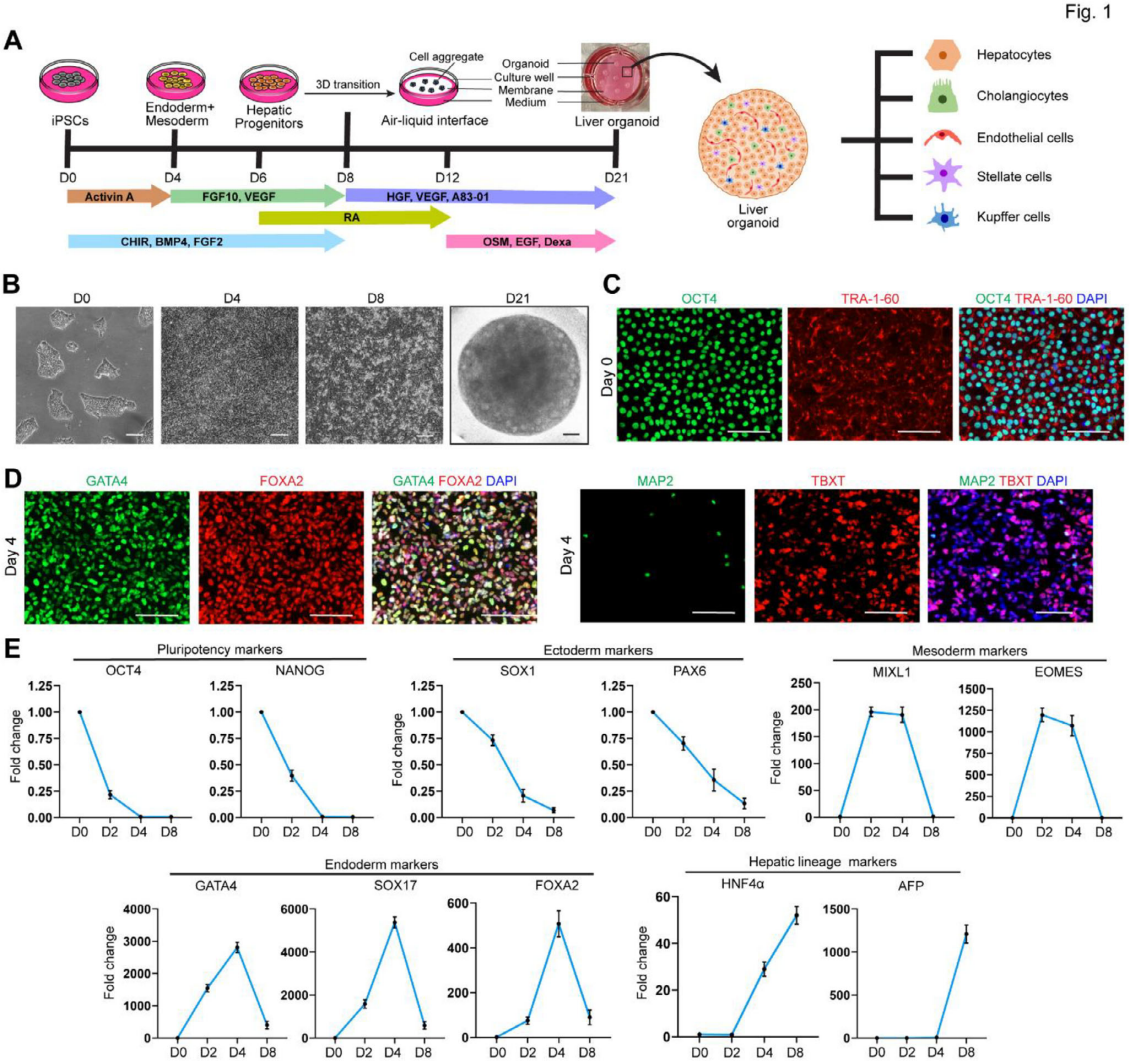
Generation of multicellular liver organoids from iPSCs: (A) Schematic representation of the differentiation procedure. (B) Phase‐contrast images show sequential morphological changes of iPSC differentiation into liver organoids (Day 0–21), scale bar: 250 µm. (C) Immunofluorescence images of iPSCs show expression of pluripotency markers (OCT4 and TRA‐1‐60) scale bar: 100 µm. (D) Immunofluorescence images on day 4 demonstrate the expression of endodermal (GATA4, FOXA2) and mesodermal (TBXT) markers, with very few cells expressing the ectodermal marker (MAP2), scale bar: 100 µm. (E) Quantitative PCR analysis indicates upregulation of endodermal and mesodermal genes, along with downregulation of pluripotency and ectodermal genes by day 4. By day 8, endodermal and mesodermal gene expression also declines, while hepatic markers HNF4α and AFP increase, consistent with progressive commitment toward a hepatic lineage. Values are determined relative to *TBP* (housekeeping gene) and presented as fold change relative to the expression in day 0 iPSCs, which is set as 1. (*n* = 3). Data presented as mean ± SEM.

To reduce variability in organoid size and structure, we dissociated the monolayer cells on D8 and resuspended them in a small volume of media to form a dense slurry, which was then pipetted carefully into uniform sized droplets on the hydrophobic surface of a polycarbonate membrane filter that floated at the ALI. Cells showed the propensity to self‐organize into compact spherical clusters after 24 h of seeding at the ALI, with an average diameter of 700 ± 100 µm. Cell aggregates at ALI were cultured in organoid formation media for 4 days. On day 12, the media was switched to organoid maturation media for the next ten days. On day 21, cell aggregates formed liver organoids, 3D organotypic structures with an average diameter of 1.6 ± 0.5 mm. To confirm that organoid viability was not compromised by the larger size, a live/dead assay was performed on day 21 organoids that demonstrated that both control and MASH donor‐derived liver organoids maintained high cell viability with minimal cell death (Figure S2A,B)

### 2.2 Cellular Characterization of Liver Organoids

To dissect the cellular repertoire within our liver organoids, we performed whole‐mount immunostaining, qPCR analysis, and flow cytometry. The immunostaining revealed cytosolic expression of mature hepatic markers: ALB and SERPINA1 along with nuclear expression of hepatic transcription factor HNF4α (Figure 2A,B). Quantitative PCR analysis also showed significant upregulation of mature hepatic markers: *ALB*, *SERPINA1*, *HNF4A*, and *CYP3A4* [20] as well as markers specific for cholangiocytes, endothelial cells, stellate cells, and Kupffer cells by day 21 of differentiation (Figure 2H). Polarization is another feature of hepatocytes, which we confirmed by the expression of tight‐junction protein zona occludens‐1 (ZO‐1) and apical transporter protein multidrug‐resistance protein 2 (MRP2/ABCC2), both of which are enriched in the apical bile‐canalicular membrane [21, 22] (Figure 2B,G). Polarization was further assessed at the ultra‐structural level by transmission electron microscopy (TEM), that depicted presence of bile canaliculi‐like structures (red *) and tight junction (yellow arrowhead) complexes (Figure 2I). Ultrastructural analysis also demonstrated the presence of numerous apical microvilli on the surface of hepatocytes along with desmosome‐like structures connecting adjacent cells. Multiple mitochondria, rough endoplasmic reticulum, and occasional glycogen inclusions and lipid droplets were also evident in the cytoplasm of hepatocytes (Figure 2I). The presence of cholangiocyte‐like cells in liver organoids was detected by immunostaining for SOX9, CK19, and CFTR that were expressed especially around duct‐like luminal structures along with acetylated α‐tubulin localized on primary cilia [23, 24] (Figure 2C). Quantitative PCR analysis also showed significant upregulation of mature biliary markers *CFTR*, *AQP1*, and *SLC10A2*/*ASBT* by day 21 of differentiation [23, 24] (Figure 2H). The expression of ALCAM/CD166 and desmin (DES) by spindle‐shaped cells suggests the presence of stellate cells [25] (Figure 2A,D). Moreover, CD31 and LYVE1 expression by luminal vascular networks, confirmed the presence of liver sinusoidal endothelial‐like cells (LSECs) [26, 27] (Figure 2A,E). Image analysis of LYVE1‐positive vascular network using AngioTool software revealed that liver organoids contained vast, interconnected networks of vasculature within organoids from each iPSC line (Figure S3A,B).

**FIGURE 2:**
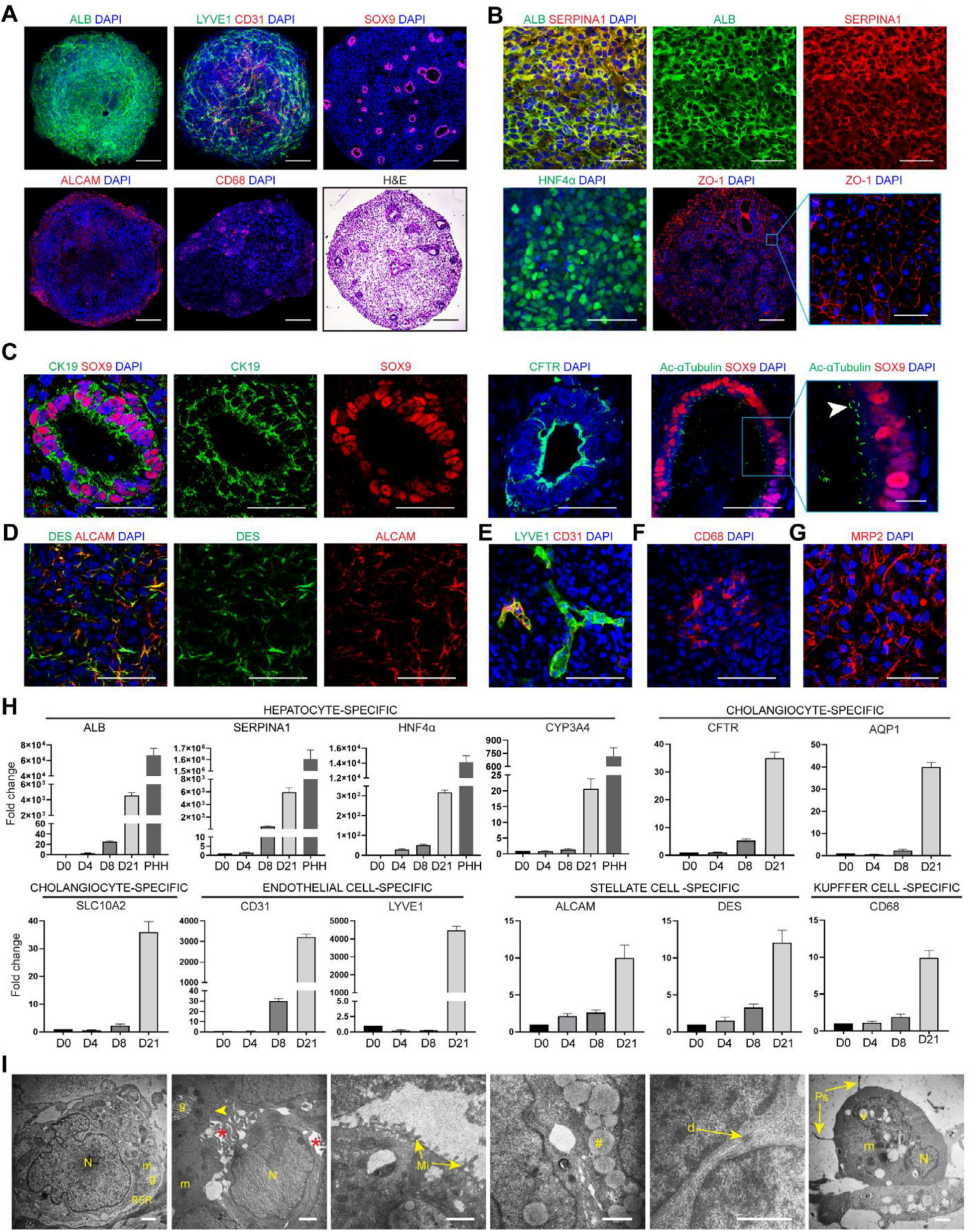
Cellular and structural characterization of liver organoids: (A) Representative immunofluorescent images of control donor‐derived liver organoids on day 21 show the expression of ALB (hepatocyte marker), LYVE1, and CD31 (endothelial marker) in whole‐mount organoids, along with histological sections showing SOX9 (cholangiocyte marker), ALCAM (stellate cell marker), and CD68 (Kupffer cell marker). A H&E stained image of a histological section from control‐donor derived liver organoid shows multiple duct‐like structures (scale bars: 500 µm). (B) Representative high magnification immunofluorescence images of whole‐mount control donor‐derived liver organoids show expression of hepatocyte markers (ALB, SERPINA1/A1AT, HNF4α), tight junction marker (ZO‐1), (C) cholangiocyte markers (CK19, SOX9, CFTR) and acetylated α‐tubulin localized on primary cilia of cholangiocytes (inset: scale bar: 10 µm), (D) stellate cell markers (DES, ALCAM), (E) endothelial cell markers (LYVE1, CD31), (F) Kupffer cell marker (CD68) (scale bars: 50 µm) (G) bile canalicular membrane marker (MRP2, scale bar: 20 µm). (H) Gene‐expression analysis of lineage‐specific markers during differentiation. Primary human hepatocytes (PHH) were used as positive control for hepatocyte‐specific markers. Values determined relative to *TBP* and presented as fold change relative to expression in day 0 iPSCs, which is set as 1 (*n* = 3). (I) Transmission electron micrographs of liver organoid showing bile canaliculi (*), tight junction (arrowhead), nucleus (N), mitochondria (m), glycogen (g), microvilli (Mi), rough endoplasmic reticulum (RER), desmosome (d), and lipid droplets (#), and a Kupffer cell showing pseudopodia (Ps) and vacuoles (v) (scale bar: 1 µm).

Importantly, whole‐mount immunostaining confirmed the presence of Kupffer‐like cells, indicated by granular cytoplasmic CD68 expression [28, 29] (Figure 2A,F), along with extending pseudopodia in TEM analysis (Figure 2I). Gene expression analysis confirmed the upregulation of endothelial (*CD31*, *LYVE1*), stellate (*ALCAM*, *DES*), and Kupffer (*CD68*) cell‐specific markers by day 21 of differentiation (Figure 2H). Hematoxylin‐Eosin (H&E) staining showed the typical structural organization, including the epithelial cells and ductal‐like structures (Figure 2A). Additionally, flow cytometric analysis further confirmed the presence of discrete cell populations expressing ALB+, CK19+, CD166+, LYVE‐1+, and CD68+ in liver organoids (Figure S4). Collectively, these observations indicate that our liver organoid model harbors epithelial as well as supportive stromal lineages, highlighting the fidelity of our differentiation protocol.

We observed that organoids could be reproducibly generated from all iPSC‐cell lines derived from MASH and control donors with no detectable variability in their differentiation efficiency, morphological characteristics, or marker expression across each iPSC donor line (Figure S5), indicating the robustness of our differentiation protocol.

To compare the ALI approach with the conventional matrigel method, we followed the same differentiation protocol, but instead of transferring cells to the air‐liquid interface on day 8, we embedded them in a matrigel drop. On day 21, matrigel‐embedded organoids displayed substantial variability in size, with diameters ranging from 300 to 800 µm, as cells self‐aggregated into variably sized structures within each matrigel drop (Figure S6A). qPCR analysis showed that matrigel‐embedded organoids expressed lower levels of hepatocyte (*ALB*), cholangiocyte (*CFTR*, *SLC10A2*), and sinusoidal‐endothelial cell markers (*LYVE1* and *CD31*), while higher levels of stellate‐cell (*DES*) and Kupffer cell (*CD68*) marker compared to ALI cultured organoids (Figure S6B). Immunofluorescence further demonstrated that matrigel‐embedded organoids expressed reduced levels of LYVE1^+^ and CD31^+^ endothelial cells (Figure S6C). Quantitative analysis of LYVE1^+^ vascular structures using AngioTool confirmed that matrigel‐embedded liver organoids developed a much sparser vascular network compared to those maintained at ALI (Figure S6D,E). A summary of differences between the ALI and matrigel‐based methods is presented in Table S1.

### 2.3 Functional Assessment of Liver Organoids

Upon differentiation, organoids acquired cellular function that is reminiscent of progressively mature hepatocytes, such as glycogen storage indicated by PAS staining, hepatocellular uptake and release of indocyanine green (ICG), and LDL uptake (Figure 3A–C). To visualize polarized epithelial cells within the bile canaliculi network, we performed a live‐imaging assay with CDFDA, a non‐fluorescent esterified form of CDF that freely diffuses into hepatocytes, is cleaved by the intracellular esterases, and is subsequently effluxed into bile canaliculi as fluorescent CDF by a transporter protein MRP2. We observed that the fluorescent CDF substrate accumulated in bile canaliculi formed between adjacent hepatocytes, thereby confirming the presence of a functional MRP2 transporter (Figure 3D). To assess the biosynthetic capacity of the liver organoids, we quantified albumin and urea secretion levels in the culture supernatant over a 24‐h period. Albumin secretion from organoids derived from control and MASH donors, as well as from primary human hepatocytes (PHH), were 6.5 ± 0.87 µg/mL/24 h, 6.1 ± 0.99 µg/mL/24 h and 8 ± 0.90 µg/mL/24 h, respectively, which were not significantly different from PHH (*p* > 0.05), indicating that organoids possess substantial biosynthetic capability (Figure 3E). Urea production was equivalent between organoids derived from control and MASH background and were substantially greater than undifferentiated iPSC controls, yet were below PHH (Figure 3F). To evaluate drug metabolism function of liver organoids, we assessed the basal and induced levels of CYP3A4 activity using luminescent substrate. After rifampicin exposure, the CYP3A4 activity was strongly induced, which was 3.9‐fold higher in treated organoids, compared with DMSO‐treated vehicle controls (Figure 3G). Since the liver is a primary site for synthesis of coagulation factors, we assessed the expression levels of *F2* (coagulation factor II), *F5* (coagulation factor V), *F7* (coagulation factor VII), *F8* (coagulation factor VIII), and *F10* (coagulation factor X) in liver organoids. Both control and MASH donor‐derived liver organoids expressed transcripts for all these coagulation factors. Notably, MASH donor‐derived liver organoids expressed elevated transcript levels of *F8* (14.17 ± 0.98 vs 6.88 ± 0.47, *p* < 0.001) and *F10* (26.2 ± 1.04 vs 16.65 ± 0.88, *p* < 0.001) compared to control donor‐derived organoids (Figure S7A). We speculate that the increased *F10* expression in MASH‐derived organoids may reflect a more fibrotic state [30], although this warrants further investigation. When compared to PHH, *F8* expression levels were elevated in both MASH (14.15 ± 0.67 vs 1 ± 0.01, *p* < 0.001) and control donor‐derived (5.62 ± 0.53 vs 1 ± 0.01, *p* < 0.001) liver organoids (Figure S7B), consistent with the understanding that this factor is predominantly produced by liver sinusoidal endothelial cells [31].

**FIGURE 3:**
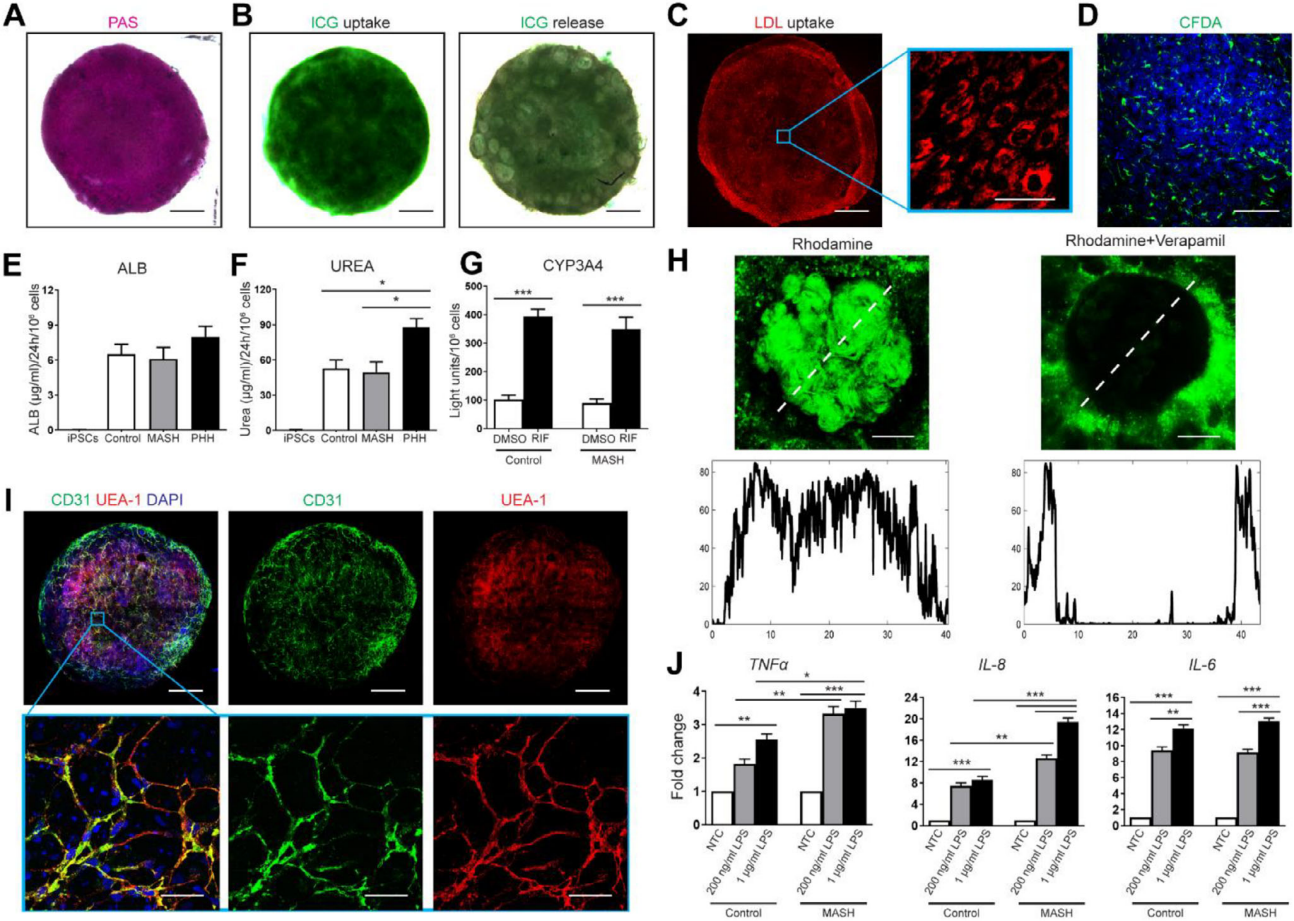
Functional characterization of different cell‐types in liver organoids: (A–G). Functional characterization of hepatocytes: (A) Representative images of whole‐mount liver organoids show PAS staining for glycogen storage, (B) ICG uptake and release, and (C) LDL uptake (scale bar: 500 µm; Inset: 50 µm). (D) Visualization of bile canaliculi network using CDFDA (scale bar: 50 µm). Production of (E) albumin and (F) urea in culture‐supernatant by day‐21 control and MASH donor liver organoids. Primary human hepatocytes (PHH) were used as positive controls, and iPSCs were used as negative controls (*n* = 4). (G) CYP3A4 activity of control and MASH donor liver organoids with or without Rifampicin (Rif) was assessed using a bioluminescent assay (*n* = 4). (H) Functional characterization of cholangiocyte‐like cells: Representative whole‐mount fluorescence images demonstrating MDR1‐dependent transport of rhodamine 123 into luminal structures and its blockage by MDR1 inhibitor, verapamil (scale bar: 10 µm). Plot profiles of intraluminal fluorescence intensity along the dotted line. (I) Functional characterization of luminal endothelial networks: Representative whole‐mount immunofluorescence images after in vitro perfusion show UEA‐1 signals co‐labeled with CD31 vascular structures (scale bar: 500 µm; Inset: 50 µm) (J) Functional characterization of Kupffer‐like cells: Quantitative PCR analysis show upregulation in gene expression of pro‐inflammatory cytokines *TNFα*, *IL‐8* and *IL‐6* in response to LPS treatment, compared to no‐treatment control (NTC). Values determined relative to *TBP* and presented as fold change relative to the expression in untreated controls, which is set as 1 (*n* = 3). Data presented as mean ± SEM. ∗*p* < 0.05, ∗∗*p* < 0.01, and ∗∗∗*p* < 0.001 (one‐way ANOVA).

The secretory potential of cholangiocyte‐like cells was confirmed using rhodamine 123 dye, a fluorescent substrate used to assess the activity of MDR1. Rhodamine 123 was actively secreted into the luminal space, and the luminal accumulation was prevented by MDR1 inhibitor, verapamil (Figure 3H), thereby confirming MDR1 functionality. Next, we assessed the presence of luminal endothelial networks using DyLight594‐conjugated UEA‐1, a lectin that selectively binds to human endothelial cells [32]. UEA‐1‐DyLight594 gradually diffused into the vascular lumen and accumulated in vascular structures. The whole‐mount immunofluorescence staining of liver organoids revealed that UEA‐1 expression overlapped with CD31+ vascular structures, thereby indicating the presence of luminal vasculature in the organoids (Figure 3I).

For Kupffer‐like cells, we treated the organoids with lipopolysaccharide (LPS), an endotoxin that binds to TLR4 receptor on Kupffer cells, triggering the production of inflammatory cytokines. Indeed, we observed a dose‐dependent increase in the gene expression of pro‐inflammatory cytokines, such as TNFα, IL‐6, and IL‐8, after treatment with LPS (Figure 3J). LPS stimulation also increased the secretion of IL‐6 and IL‐8 proteins in the cell culture supernatant, indicating that organoids were responsive to inflammatory stimuli (Figure S8). Taken together, these results support that our liver organoid model exhibits lineage‐specific functionality of hepatocytes, cholangiocytes, endothelial, and Kupffer cells.

### 2.4 Free‐Fatty Acid Exposure Triggers Inflammatory and Fibrotic Responses in Liver Organoids

Given the presence of hepatocyte, stellate, and Kupffer‐like cells in our liver organoid model, we sought to investigate if MASH and control donor‐derived liver organoids exhibited differences in steatosis, inflammatory, and fibrotic responses to free‐fatty acid (FFA) exposure. To determine an effective dose for inducing steatosis, liver organoids were exposed to 25, 50, or 100 µM oleic acid (OA), a common dietary fatty acid for 24 h, and BODIPY staining was performed to visualize the lipid droplet accumulation. A 100 µM OA concentration produced a robust steatotic response in both control and MASH‐donor derived liver organoids (Figure S9), and this dose was utilized for all subsequent experiments.

Following treatment with 100 µM OA, both MASH and control donor‐derived liver organoids exhibited increased lipid accumulation in response to OA treatment; however, higher lipid deposition with prominent macro‐vesicular steatosis was observed in MASH donor‐derived liver organoids, highlighting their increased susceptibility to lipid accumulation (Figure 4A and Figure S10A for each iPSC line). To assess whether liver organoids exhibited a fibrotic response to OA treatment, alterations in ECM components were evaluated. Immunofluorescence analysis revealed increased expression of COL‐1 (ECM marker) and ACTA2 (activated stellate cell marker), following OA treatment and relative to BSA control, in both control (COL‐1: 14.43 ± 1.88 vs. 4.5 ± 1.25; *p* < 0.01, ACTA2: 3.86 ± 0.42 vs. 1.77 ± 0.38; *p* < 0.05) and MASH donor liver organoids (COL‐1: 24.73 ± 1.70 vs. 17.13 ± 1.48; *p* < 0.05, ACTA2: 8.63 ± 0.54 vs. 4.50 ± 0.50; *p* < 0.001), suggesting the presence of activated stellate‐like cells that contribute to increased ECM deposition. Notably, COL1A1 and ACTA2 expression levels were significantly higher in OA‐treated MASH organoids compared to OA‐treated controls (COL‐1: 24.73 ± 1.70 vs. 14.43 ± 1.88; *p* < 0.01, ACTA2: 8.63 ± 0.54 vs. 3.86 ± 0.42; *p* < 0.001), suggesting an exacerbated fibrotic phenotype in the disease model (Figure 4B and Figure S10B for each iPSC line). Picrosirius red staining further confirmed significant deposition of collagen and fibrosis in OA‐treated MASH organoids compared to OA‐treated control organoids (27.75 ± 2.18 vs. 18.00 ± 2.27; *p* < 0.05 (Figure 4C). In agreement with this, OA treatment of MASH organoids also led to increased gene expression of fibrosis associated genes: *TGF‐β* (1.77 ± 0.06 vs. 1.41 ± 0.06; *p* < 0.01) and *COL1A1* (1.97 ± 0.11 vs. 1.39 ± 0.06; *p* < 0.001), and pro‐inflammatory markers: *TNFα* (2.57 ± 0.08 vs. 2.1 ± 0.14; *p* < 0.05) and *IL‐23* (2.33 ± 0.12 vs. 1.83 ± 0.07; *p* < 0.01), compared to OA treated control organoids (Figure 4D). ELISA analysis further confirmed that OA‐treated MASH organoids secreted higher levels of fibrosis‐related proteins, such as pro‐collagen 1 (6887 ± 1231 ng/mL vs. 1165 ± 102.2 ng/mL; *p* < 0.001), ACTA2 (3.21 ± 0.12 ng/mL vs. 2.63 ± 0.08 ng/mL; *p* < 0.01), TGF‐β1 (9.91 ± 0.65 pg/mL vs. 4.99 ± 0.57 pg/ml; *p* < 0.001), as well as inflammatory cytokines like IL‐8 (122.3 ± 8.48 pg/mL vs. 39.63 ± 3.67 pg/mL; *p* < 0.001) and IL‐23 (6.86 ± 0.43 pg/mL vs. 5.38 ± 0.32 pg/mL; p = 0.15, n.s), compared to OA‐treated control organoids (Figure 4E). Overall, these results indicate that MASH donor‐derived liver organoids appear to be more susceptible to OA treatment exhibiting prominent macro‐vesicular steatosis and increased inflammatory and fibrotic responses.

**FIGURE 4:**
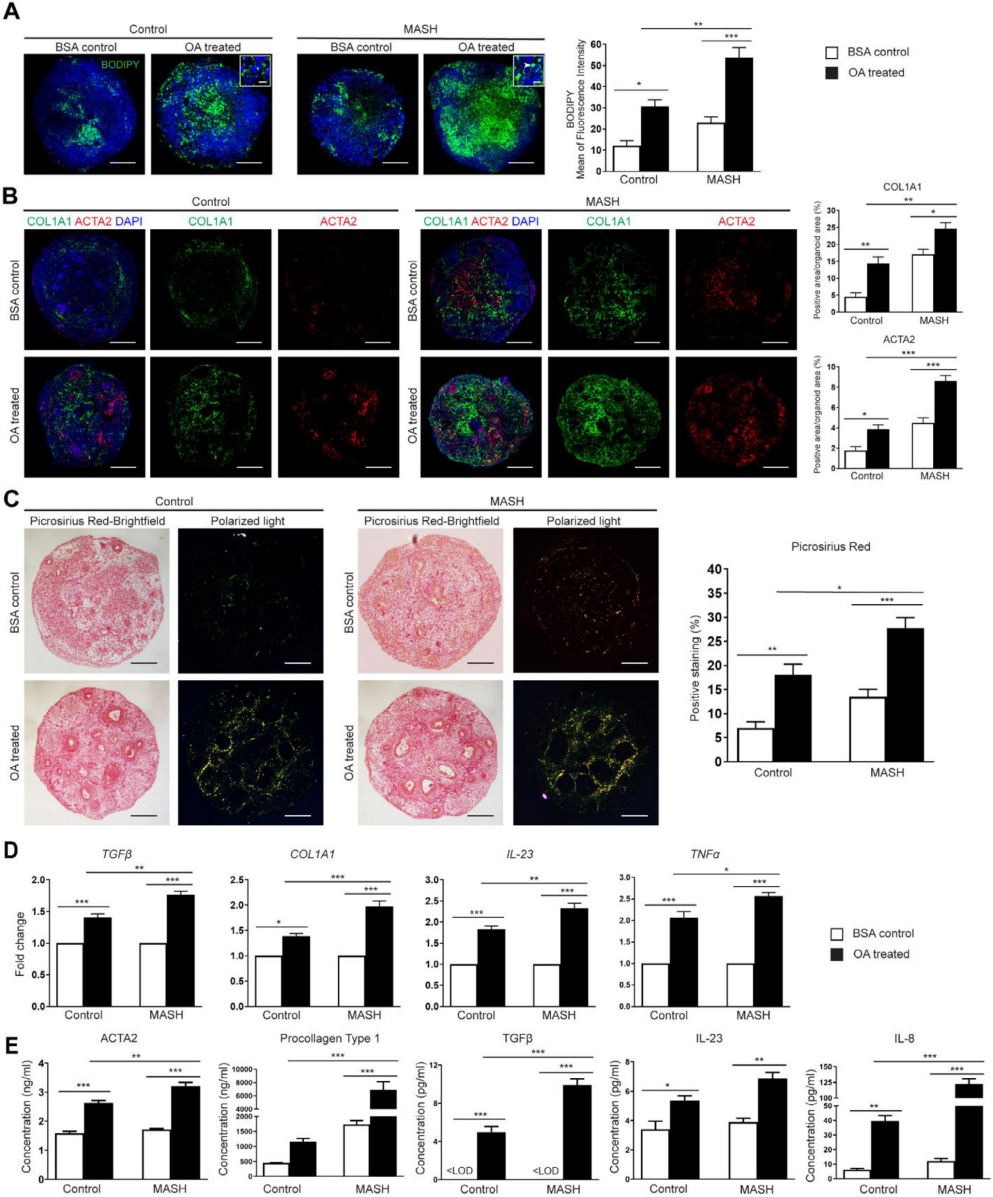
Modeling steatohepatitis pathology using liver organoids: (A) Representative whole‐mount BODIPY staining images show accumulation of neutral lipid droplets in oleic acid (OA) and BSA (vehicle‐control) treated MASH and control‐donor liver organoids; scale bar: 500 µm (Inset: white arrowhead showing macro‐steatosis; scale bar: 10 µm). Quantification of BODIPY fluorescence intensity using ImageJ (*n* = 6). (B) Representative immunofluorescent images of histological sections from control and MASH donor‐derived liver organoids, show the expression of fibrosis markers COL1A1 and α‐SMA following OA treatment, with quantification of the % positive area using ImageJ (*n* = 6). (C) Representative images of histological sections stained with picrosirius red, shown under bright‐field and polarized light, from MASH and control‐donor liver organoids treated with OA or BSA, with quantification of the % positive staining using ImageJ (*n* = 6, scale bar: 500 µm). (D) Quantitative PCR analysis depicts gene expression of fibrosis associated genes *TGFβ* and *COL1A1* and pro‐inflammatory cytokines *IL‐23* and *TNFα* in response to OA treatment in MASH and control liver organoids. Values determined relative to *TBP* and presented as fold change relative to the expression in BSA controls, which is set as 1 (*n* = 4). (E) ELISA‐based quantification of fibrosis‐related proteins (ACTA2, procollagen Type 1, and TGFβ) and inflammatory cytokines (IL‐23 and IL‐8) in cell‐culture supernatants from control and MASH liver organoids following oleic acid (OA) treatment (*n* = 4, <LOD: below limit of detection). Data presented as mean ± SEM. ∗*p* < 0.05, ∗∗*p* < 0.01, and ∗∗∗*p* < 0.001 (one‐way ANOVA).

### 2.5 MASH Donor‐Derived Liver Organoids Exhibit Altered Lipidomic Profile

Accumulating evidence suggests that MASH is associated with global changes in the composition of hepatic lipid species [33]. This prompted us to compare the hepatic lipid profile of MASH and control donor‐derived liver organoids. We observed a higher level of triacylglycerol (TG) and cholesteryl esters (CE) lipids and a lower level of phospholipids in MASH donor‐derived organoids compared to control organoids (Figure 5).

**FIGURE 5:**
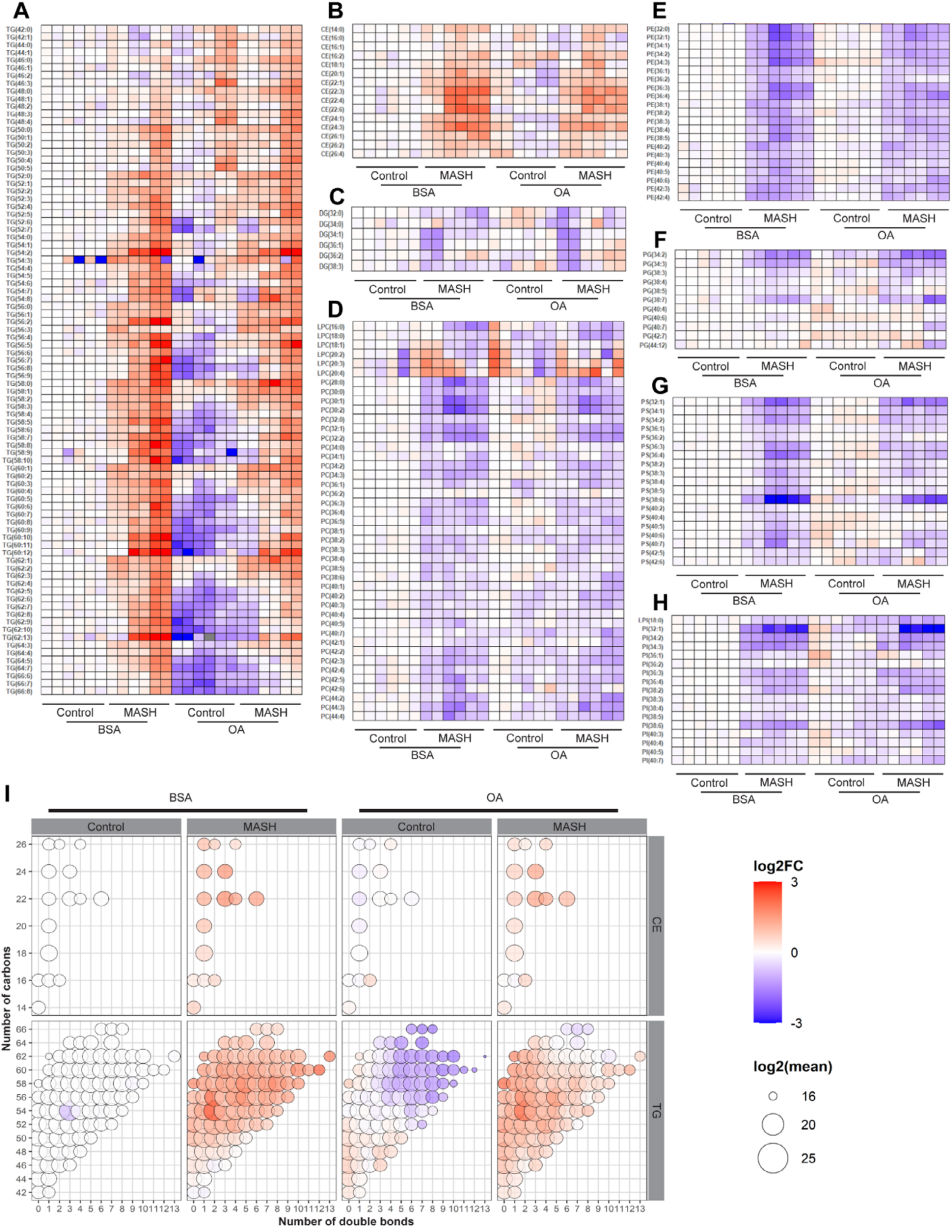
MASH and oleic acid treatment alter organoid lipidomes: (A–H) The relative abundance levels of each lipid relative to the level in untreated organoids derived from healthy donors. Each column represents a single organoid. (I) Bubble plot of individual CE and TG species. Each species is shown as log2 relative fold change to untreated healthy control (color) and log2 mean of the ion counts (bubble size). Abbreviations: TG, triglyceride; CE, cholesteryl esters; DG, diglyceride; LPC, lysophosphatidylcholine; PC, phosphatidylcholine; PE, phosphatidylenthanolamine; PG, phosphatidylglycerol; PS, phosphatidylserine; LPI, lysophosphatidylinositol; PI, phosphatidylinositol, *n* = 6.

Next, we investigated the impact of OA treatment on the organoids. OA treatment to control donor‐derived liver organoids caused a broad impact on the lipidome. Shorter chained TGs or those with less than 2 double bonds were increased by OA treatment, while those with two or more double bonds were reduced (Figure 5A,I). Compared to TGs, OA treatment had less effect on the other classes of lipids (Figure 5B,C). Some phospholipids with shorter tails, i.e., those with a sum total of 32 or fewer carbons in the two tails were elevated, while those with a greater number of carbons were reduced (Figure 5C–H). Since we observed that OA treatment altered the lipids in organoids from control donors, we determined the impact of OA treatment on organoids derived from MASH donors. Relative to untreated organoids from control donors, OA treatment in MASH donor‐derived organoids increased the levels of TG and CE lipids (Figure 5A,B,I). Similar to untreated MASH organoids, OA treated MASH organoids also exhibited reduced phospholipid levels (Figure 5D–H).

To investigate whether lipidomic signatures captured in our organoids resemble lipidomic observation in MASLD/MASH liver biopsies, we compared our lipidomic results to two recent studies that examined liver tissue collected from patients: Vvedenskava et al. (49 healthy donors and 94 MASH donors) and Collin de l'Hortet et al. (3 healthy and 3 diseased donors [33, 34]). In general, our lipidomic approach identified a greater number of lipids, likely due to greater lipid coverage by our LC‐MS/MS lipidomic methods. Nonetheless, several lipids increased or decreased in MASH donor‐derived liver organoids were also altered in the biopsy samples relative to healthy liver biopsy samples (Figure S11A,B). Of the 20 lipids increased in MASH biopsies identified by Vvedenskaya et al., 12 were increased in OA‐treated MASH donor‐derived liver organoids (Figure S11C,D). Of the 55 lipids decreased in our study, 25 were also decreased in the liver biopsy studies. Finally, the Vvedenskava et al. liver biopsy study had a greater level of agreement with the changes observed in the organoids than the Collin de l'Hortet et al. study. Overall, these observations suggest that MASH globally shifted the lipidome of organoids in ways similar to lipidomic changes observed in human liver biopsies.

### 2.6 Liver Organoids Enable Modeling of Drug‐Induced Toxicity

We further assessed the utility of liver organoids to predict drug‐toxicity using acetaminophen (APAP) as a prototype drug that is metabolized primarily in the liver. To evaluate hepatotoxicity in our liver organoid model, liver organoids were treated with 5, 25, or 50 mmol/L APAP for 3 days. APAP treatment induced a significant, dose‐dependent increase in TUNEL‐positive apoptotic cells, with markedly elevated apoptosis observed at a 50 mmol/L dose (Figure S12); therefore, we utilized 25 mmol/L APAP dose for subsequent experiments.

Growing evidence suggests that APAP‐induced hepatotoxicity is more severe in MASLD patients [35]. To model this condition, we first pretreated our organoids with oleic acid (100 µmol/L) to induce steatosis, followed by 25 mmol/L APAP treatment. We then analyzed the gene expression of glutamate‐cysteine ligase catalytic subunit (*GCLC*) and glutamate‐cysteine ligase modifier subunit (*GCLM*), that play an essential role in scavenging reactive oxygen species (ROS) by catalyzing the initial rate‐limiting step for glutathione (GSH) synthesis [36]. The gene expression analysis revealed that both *GCLC* and *GCLM* were significantly decreased in the OA + APAP treated group, compared to APAP only or no treatment control groups. The differences were more significant in OA + APAP treated MASH donor organoids compared to OA + APAP treated control organoids (GCLC: 0.28 ± 0.03 vs 0.44 ± 0.03, *p* < 0.05; GCLM: 0.25 ± 0.03 vs 0.43 ± 0.03, *p* < 0.05, Figure 6A). Additionally, the gene expression levels of antioxidant enzymes: glutathione peroxidase 1 (*GPX1*) and NAD(P)H: quinone oxidoreductase (*NQO1*) were also significantly decreased in the OA + APAP treated group, compared to APAP only or no treatment control groups, demonstrating that OA pre‐treatment leads to lower antioxidant capacity. With respect to pro‐inflammatory markers, the expression of *TNF‐α* was significantly increased in the OA + APAP treated group, compared to APAP only or no treatment control groups, with even greater elevation in the OA + APAP treated MASH organoids compared to OA + APAP treated control organoids (23.64 ± 1.22 vs 17.38 ± 0.89, *p* < 0.05, Figure 6A).

**FIGURE 6:**
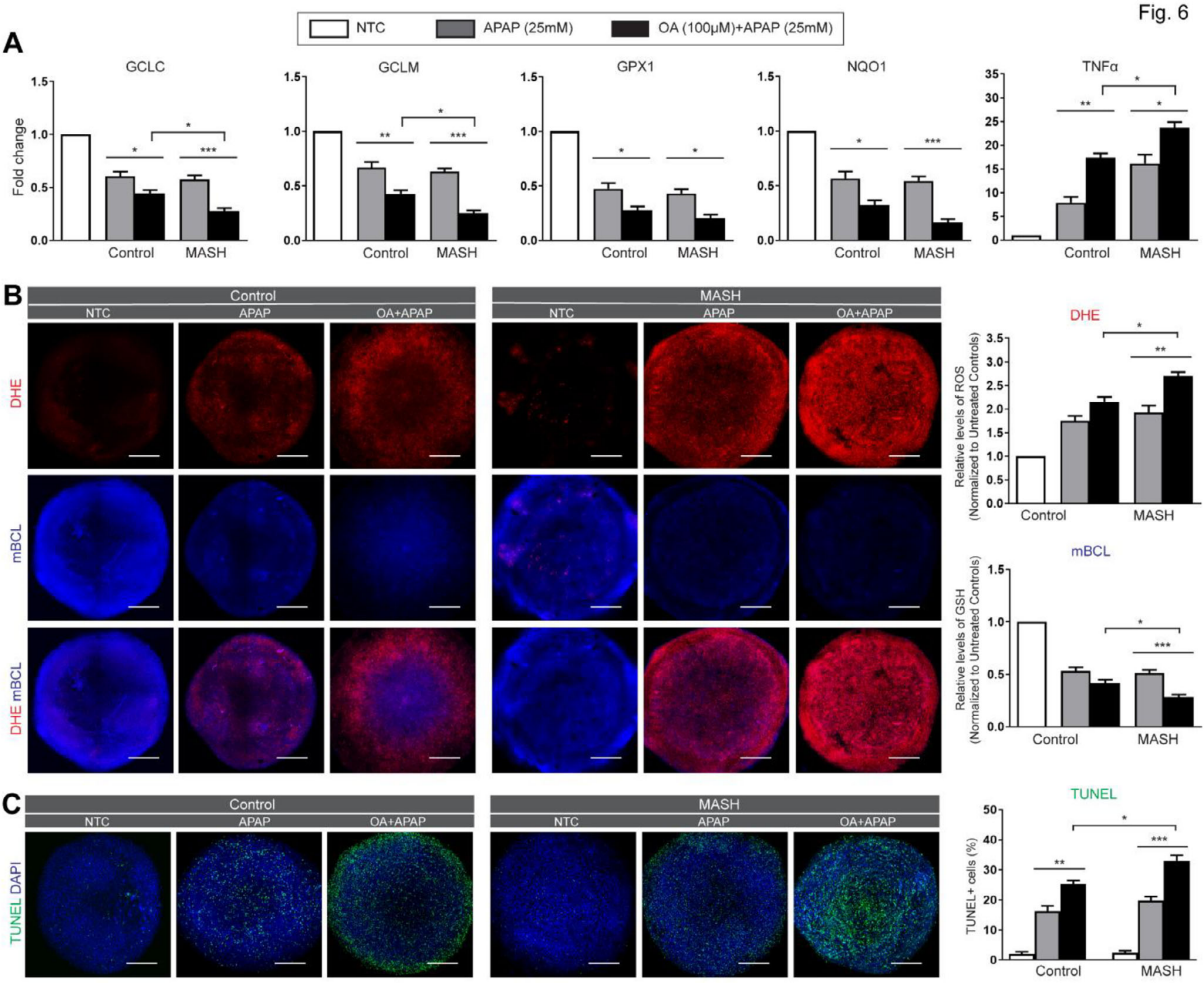
Modeling drug‐induced toxicity using liver organoids: (A) Quantitative PCR analysis of *GCLC, GCLM*, *GPX1*, *NQO1*, and *TNFα* showing the effect of oleic acid pre‐treatment on APAP induced cytotoxicity in control and MASH liver organoids. Values determined relative to *TBP* and presented as fold change relative to the expression in untreated controls, which is set as 1 (*n* = 6). Data presented as mean ± SEM. (B) Representative whole‐mount fluorescence images of control and MASH donor liver organoids treated with 25 mM APAP alone or with 100 µM OA, showing reactive oxygen species (ROS) formation and glutathione (GSH) content using dihydroethidium (DHE) and monochlorobimane (mBCL), respectively, and (C) apoptosis using TUNEL staining (scale bar: 500 µm). Graphs depict ImageJ quantification of relative level of ROS by DHE, relative level of GSH by mBCL, and apoptosis by TUNEL^+^ cells. Data presented as mean ± SEM (*n* = 6). ∗*p* < 0.05, ∗∗*p* < 0.01, and ∗∗∗*p* < 0.001 (one‐way ANOVA).

Next, we analyzed the levels of ROS production (DHE) and GSH adducts (mBCL). We found that GSH levels (mBCL) were significantly decreased, while ROS production (DHE) was significantly increased, in the OA + APAP treated group, compared to APAP only or no treatment control groups. The differences were even more pronounced in the MASH background where the OA + APAP treated MASH donor organoids were compared to OA + APAP treated control donor organoids (mBCL: 0.28 ± 0.02 vs 0.42 ± 0.03, *p* < 0.05; DHE: 2.7 ± 0.08 vs 2.2 ± 0.10, *p* < 0.05) (Figure 6B). Furthermore, the number of TUNEL‐positive cells was significantly higher in the OA + APAP treated group compared to APAP only or no treatment control groups. Notably, when comparing the MASH organoids to the controls, the OA + APAP‐treated MASH group exhibited a significantly higher number of TUNEL‐positive cells than the OA + APAP‐treated control organoids (33 ± 1.83 vs 25.25 ± 1.25, *p* < 0.05, Figure 6C). These data indicate that OA pretreatment exacerbates APAP induced hepatotoxicity in liver organoids generated from iPSCs with a MASH background.

Since mitochondria is the critical target during APAP‐induced hepatotoxicity [37], we assessed mitochondrial ROS levels, changes in the mitochondrial membrane potential, and adenosine triphosphate (ATP) production following APAP and OA + APAP treatment. Mitochondrial ROS levels were analyzed using the superoxide probe MitoSOX Red, which showed that mitochondrial ROS levels were significantly increased in the OA + APAP treated group, compared to APAP only or no treatment control groups. This increase was even more pronounced in the MASH background, where the OA + APAP treated MASH donor organoids exhibited substantially higher mitochondrial ROS levels than OA + APAP treated control donor organoids (2.21 ± 0.06 vs 1.87 ± 0.04, *p* < 0.05) (Figure S13A,C). Next, JC‐1, a dual‐emission lipophilic cationic dye was used to indirectly evaluate changes in the mitochondrial membrane potential (MMP). After entering the cells, the dye accumulates within mitochondria in a potential‐dependent manner, producing red fluorescence for highly energetic mitochondria and green fluorescence in those with reduced membrane potential. Both the APAP and OA + APAP treated groups showed a significant reduction in the red‐to‐green fluorescence ratio, indicating reduced MMP, while the no treatment control (NTC) group maintained a higher ratio in both control (NTC vs APAP: 2.91 ± 0.16 vs 0.85 ± 0.05, *p* < 0.001 and NTC vs OA + APAP: 2.91 ± 0.16 vs 0.65 ± 0.05, *p* < 0.001) and MASH donor‐derived liver organoids (NTC vs APAP: 2.68 ± 0.18 vs 0.82 ± 0.06, *p* < 0.001 and NTC vs OA + APAP: 2.68 ± 0.18 vs 0.53 ± 0.04, *p* < 0.001) consistent with high mitochondrial activity (Figure S13B,D). Intracellular ATP levels were measured to assess the mitochondrial bioenergetics. Both APAP and OA + APAP treated groups exhibited significantly reduced levels of ATP compared to the NTC group in liver organoids derived from both control (NTC vs APAP: 6.80 ± 0.20 vs 2.87 ± 0.35, *p* < 0.001 and NTC vs OA + APAP: 6.80 ± 0.20 vs 2.48 ± 0.29, *p* < 0.001) and MASH donors (NTC vs APAP: 5.06 ± 0.45 vs 2.38 ± 0.26, *p* < 0.001 and NTC vs OA + APAP: 5.06 ± 0.45 vs 1.75 ± 0.26, *p* < 0.001), indicating that APAP exposure impairs mitochondrial function and leads to depletion of the cellular ATP reserves (Figure S13E). Taken together, this highlights the potential that liver organoids can be used as a pre‐clinical tool to predict potential “vulnerability” to drug‐induced liver injury in patients with pre‐conditions such as MASLD or obesity.

### 2.7 Liver Organoids are Transplantable

To assess the suitability of liver organoids for in vivo use, we engrafted control‐donor derived liver organoids under the kidney capsule of NSG mice (Figure 7A). We preferred the renal subcapsular space as an engraftment site because it is highly vascularized, readily accessible, and transplanted materials are easy to identify upon retrieval [38]. After four weeks post‐transplant, the graft exhibited extensive growth, with prominently visible blood vessels (Figure 7B). To ascertain if the cells were of human origin in the graft, we stained tissues with human nuclear antigen (HuNu) and human nuclear mitotic apparatus protein (NuMA). Interestingly, human‐specific CD31+ vascular networks invaded host tissue and extended into the recipient mouse kidney parenchyma. Murine‐specific CD31+ vessels emanating from the host kidney also invaded the graft (Figure 7C). Further, mouse erythrocytes were detected in human vascular networks, suggesting a patent vascular anastomosis between the graft and host vasculature (Figure 7C). Sprouting of LYVE1 expressing LSECs was also observed in the graft (Figure 7C). Next, we identified large hepatic clusters expressing human ALB and SERPINA1 (Figure 7D), stellate cells expressing DES (Figure 7E), Kupffer cells expressing CD68 (Figure 7F), and biliary duct‐like structures expressing CFTR, CK19, and CK7 (Figure 7G). Human‐specific serum albumin levels were assessed as a read‐out for functional engraftment, which showed that engrafted liver organoids secreted human albumin into the bloodstream of recipient mice (538 ± 95.86 ng/mL at 2 weeks and 1076 ± 171.80 ng/mL at 4 weeks), whereas no human albumin was detected in sham‐operated control mice (Figure 7H). These results demonstrate that liver organoids are amenable to transplantation showing survival, viability, vascularization, and preserved function.

**FIGURE 7:**
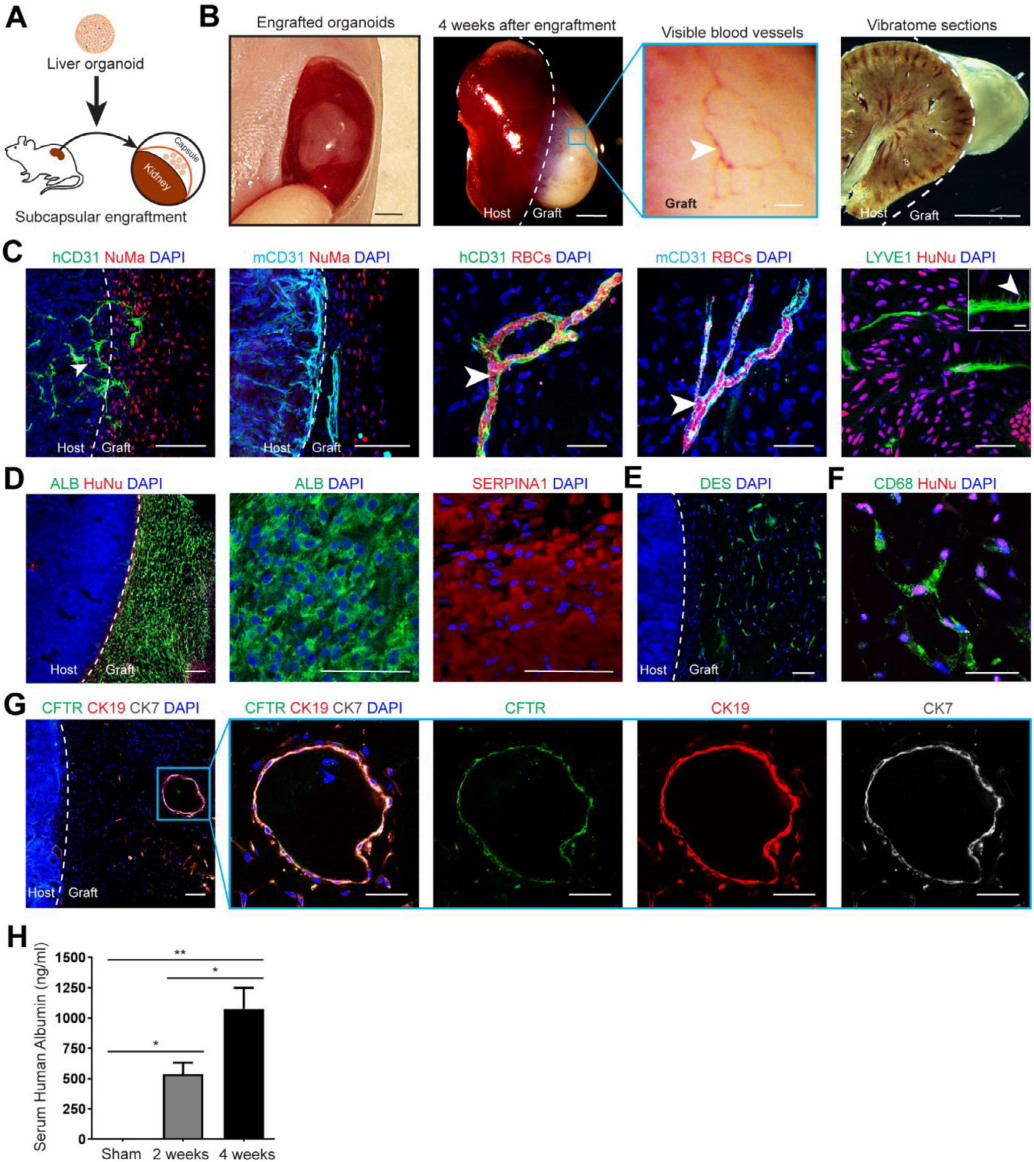
In vivo engraftment of liver organoids. (A) Schematic depicts renal subcapsular engraftment of liver organoids under the murine kidney capsule. (B) Image shows liver organoids immediately after implantation, and four weeks post‐engraftment with visible blood vessels (inset: white arrowhead, scale bar: 200 µm). Stereomicroscope image shows a vibratome section of mouse kidney with graft; scale bars: 1 mm. (C) Low magnification immunofluorescence images show human NuMa+ CD31+ endothelial networks entering the host kidney and mouse CD31+ endothelial networks entering the graft (scale bar: 100 µm). High magnification images show mouse erythrocytes (arrowhead) within both human and mouse CD31+ endothelial vessels, and HuNu+ LYVE1+ liver sinusoidal endothelial cells show sprouting (inset: arrowhead; scale bar: 10 µm) (scale bars: 50 µm). (D) Representative immunofluorescence images show ALB+ and SERPINA1+ hepatic clusters (scale bar: 100 µm), (E) DES+ stellate cells (scale bar: 100 µm), (F) CD68+ Kupffer cells (scale bar: 50 µm), and (G) CK19+, CK7+, and CFTR+ biliary structures, in the graft (scale bar: 100 µm; inset: 50 µm). (H) Quantification of human albumin levels in mouse serum by ELISA after 2 and 4 weeks of engraftment. Data presented as mean ± SEM. (*n* = 6). ∗*p* < 0.05, ∗∗*p* < 0.01, and ∗∗∗*p* < 0.001 (one‐way ANOVA).

## 3 Discussion and Conclusion

In this study, we report a simple and efficient stepwise strategy to generate liver organoids from iPSCs that contain parenchymal (hepatocytes and cholangiocytes) and non‐parenchymal cell populations (endothelial, stellate, and immune cells). Additionally, generation at ALI, a matrix‐free environment, enables easy retrieval of in vitro generated organoids for downstream applications. Our protocol reproducibly generated organoids in a relatively short duration (∼21 days) from all MASH and control donor iPSCs without any significant differences in differentiation capacity. To the best of our knowledge, this is the first study reporting a successful generation of in situ multicellular functional liver organoids from control and MASH donor iPSCs, without the need for exogenous cells, genetic manipulation or Matrigel/ECM embedding.

Current methods for generating liver organoids rely on embedding within Matrigel or other ECM substrates to support or modulate the organoid micro‐environment [5, 6, 10, 11, 13, 39, 40, 41, 42]. However, Matrigel presents challenges, including lot‐to‐lot variability that can affect cellular fate decisions and function. Additionally, its xenogeneic origin limits use in clinical settings [43]. Few efforts have been made to establish multicellular liver organoids using ECM independent approaches. Harrison et al. generated multicellular functional liver organoids with a liver‐like cellular repertoire in ECM‐free suspension culture; however, they did not demonstrate whether their approach is compatible with differentiating patient‐derived iPSCs for modeling human disease [15]. Weng et al. generated liver organoids using rotating wall vessels, but their study did not provide a comprehensive characterization of the major hepatic cell populations [14]. Beyond these limitations, suspension‐based approaches inherently face difficulties in controlling cell number per organoid and regulating organoid size, as the cells self‐organize into aggregates of variable sizes. Wang et al. modeled non‐alcoholic fatty liver disease on‐a‐chip using organoids composed of hepatocytes and cholangiocytes, however, this simplified system lacked Kupffer cells and hepatic stellate cells, which are essential for capturing the inflammatory and fibrotic responses that drive disease pathogenesis [44]. More recently, Saiki and colleagues reported a method to generate human liver bud organoids by individually differentiating iPSCs into hepatic endoderm, mesenchyme, and endothelial progenitors. These individually differentiated cell populations were then subsequently mixed in matrigel and cultured on an inverted multilayered air‐liquid interface (IMALI) to study interactions between hepatic and endothelial subpopulations [45]. While this strategy leveraged the organotypic advantages of ALI culture, reliance on matrigel persists as a translational barrier. Here, we establish a matrigel‐free ALI platform that provides a physiologically relevant 3D microenvironment, enabling adequate oxygenation from the surface and nutrient delivery from the bottom media. This environment enhances cell‐cell interactions and enables differentiating cells to self‐organize into organotypic structures [46, 47].

Furthermore, many reported organoid models are generally simplistic, composed of hepatocytes or cholangiocytes only, rendering them less physiologically relevant compared to the native liver tissue [5, 8, 10, 39, 41, 48]. Alternatively, other approaches for generating multicellular liver organoids involve combining epithelial and supportive cell lineages, which may have been differentiated individually from iPSCs or sourced from various postnatal origins [6, 9, 40, 45, 49]. However, such strategies may increase the risk of immune rejection or sensitization to host antigens in transplant settings and pose challenges in optimizing culture conditions to support the maintenance of multiple cell types simultaneously. To address these concerns, our differentiation approach promoted simultaneous induction of iPSCs into endodermal and mesodermal lineages, which eventually differentiated into hepatic, biliary, stellate, endothelial, and Kupffer‐like cells. This coordinated differentiation recapitulates key aspects of embryonic liver development, where lineage specification is driven by temporally integrated signaling cues. WNT activation via GSK3 inhibitor CHIR99021 facilitated the transition of iPSCs from pluripotency to the primitive streak stage, which exhibited lineage‐specific responsiveness to BMP4, promoting mesodermal differentiation, and to Activin A, inducing endodermal specification. Lineage specification was validated at day 4 by quantitative PCR and immunocytochemical analysis, confirming the successful induction of endodermal and mesodermal populations and the absence of ectodermal differentiation. To mimic the inductive signals from the cardiac mesoderm and septum transversum mesenchyme, FGF and BMP signaling was activated to promote foregut restriction and hepatic lineage commitment. Co‐differentiation and subsequent maturation into epithelial and mesenchymal lineages was supported by organoid formation and organoid maturation media containing RA, which plays a key role in specifying both parenchymal and non‐parenchymal hepatic cell‐types [50, 51, 52], along with A83‐01, HGF, OSM and dexamethasone as hepatic inducers [53, 54, 55], as well as mitogenic inducers like VEGF to promote endothelial cell differentiation and maturation [27, 56], and EGF to drive biliary differentiation and maturation [57]. Gene‐expression profiling and flow cytometric assessment on day 21 validated the successful differentiation and maturation of different hepatic cell‐types. An important aspect of this study is the single culture condition that supports differentiation of all hepatic cell‐types together, eliminating the requirement of exogenous cells. This strategy reduces experimental variability and, most importantly, lowers the risk of immune rejection for potential transplant applications.

The generation of liver organoids with luminal vasculature remains a challenge. However, few studies have successfully generated liver organoids with endothelial networks but have demonstrated the development of functional luminal vasculature only after in vivo transplantation, suggesting the role of angiogenic cues derived from the host tissue to drive vasculogenesis [6, 40, 58, 59]. In contrast, our protocol successfully generated liver organoids with lumenized vessels that was confirmed by detection of fluorescent UEA‐1 in CD31 expressing vascular lumens. The organoids also exhibited structural features resembling bile‐canaliculi‐like network and lineage‐specific functionality of different cell‐types. However, further studies using single‐cell RNA sequencing (scRNA‐seq) and functional network analysis would be necessary to allow a better understanding of functional association and zonal identity of different cell‐types.

The generation of iPSCs from patient‐derived somatic cells has revolutionized disease modeling and drug discovery. Previous studies employing MASLD/MASH patient iPSCs have shown that hepatocyte‐like cells differentiated from MASLD/MASH donors exhibited increased lipid accumulation compared to healthy controls, along with a distinct transcriptomic signature consistent with MASLD pathology [60, 61, 62]. Since MASLD is a complex disease where cellular crosstalk is critical for disease progression [63], organoids derived from patient iPSCs with the full complement of liver cells will be useful for modeling such progressive disease. Given the presence of epithelial and supportive lineages in our liver organoids, we investigated differences in the responses of MASH and control donor‐derived liver organoids to free‐fatty acid exposure and APAP treatment. MASH donor‐derived liver organoids exhibited increased susceptibility to OA induced stress and acetaminophen‐induced toxicity. This observation is likely attributable to the preserved epigenetic memory of iPSCs from the diseased donor [64, 65, 66], which manifested upon differentiation, however, comprehensive analysis of their epigenetic landscape will be necessary to confirm this. Overall, our study suggests the feasibility of using liver organoids to model liver diseases and drug‐toxicity. To strengthen predictive reliability for drug toxicity, efficacy, and disease modeling, future studies should incorporate a wider range of patient cell‐lines to address patient‐to‐patient variability.

Excessive intracellular accumulation of neutral lipids is a metabolic hallmark of MASLD/ MASH [33]. We compared the lipidomic profile of MASH and control donor‐derived liver organoids and found that MASH organoids exhibited increased levels of TG and CE and decreased levels of phospholipids, such as PE, PC, PS, and PI compared to control organoids. This observation is consistent with the lipidomic analysis of the MASH patient liver biopsies [33, 67], which indicates that patient‐derived liver organoids do recapitulate changes in their lipidome as seen in patients, even in the absence of fatty acid supplementation. However, there was less impact on DG lipids in MASH liver organoids, which may depend on the dose or type of dietary free‐fatty acid used to induce steatosis. We also investigated the effect of OA treatment on the lipidome and found that OA treatment had a significant effect on the levels of TGs. OA treatment in MASH donor derived organoids led to an increase in TGs with shorter chains and few to no double bonds. OA treatment in control healthy donor derived organoids led to a decrease in the level of TGs with longer chains and polyunsaturated double bonds. These results are likely to be due to the shift in the FA pool toward a greater number of free C18:1 (OA) and its downstream metabolites available for lipid synthesis [68]. Our observations corroborate with previous lipid changes observed in liver biopsies, with a majority of lipids observed to be increased in MASH also being upregulated in OA treated MASH donor derived organoids [33, 34]. In addition to lipids in the liver, MASH also shifts the serum lipid profile. While serum and cellular profiles are different due to the nature of their forms (e.g., lipoprotein particles in serum and lipid droplets or membranes in cells), our observation that shorter chain TGs with fewer double bonds were increased in MASH organoids is similar to the serum lipid changes reported in patients with MASH or advanced fibrosis [69, 70].

For successful generation of clinically transplantable liver tissues, functional vascularization is important for graft survival and function. We observed graft‐derived human CD31+ vessels invaded the mouse kidney parenchyma and anastomose with the host vasculature with evident erythrocytes. Although anastomosis of the host and graft vessels have been demonstrated [6, 7], this is the first time we are showing anastomosis with evident mouse erythrocytes within the human‐derived vascular lumen. Interestingly, hepatic and biliary structures, along with LSECs, stellate and Kupffer‐like cells, were also well‐maintained following engraftment, with the detection of human albumin in mouse bloodstream, further confirming successful engraftment.

In conclusion, here we demonstrated the generation of human liver organoids from control and diseased donor‐derived iPSCs with concurrent differentiation into different cell‐types. Liver organoids exhibited the potential to model liver disease, drug sensitivity and hepatoxicity, and engraftment capability in vivo. However, further biochemical, transcriptomic, and proteomic profiling across different cell lines would be critical for understanding the cellular contributions to disease development. We envisage that these organoids can serve as a powerful tool for pathological and developmental research in the liver and as a promising cell‐source for autologous transplantation and personalized medicine.

## 4 Experimental Section

### 4.1 Human Pluripotent Stem Cell‐Culture

Deidentified human iPSC cell lines derived from control (CW10192, CW10021) and MASH donors (CW10166, CW10183) were purchased from the California Institute of Regenerative Medicine (CIRM) iPSC repository. All iPSC cell lines were maintained on Matrigel (Corning) coated dishes in mTESR1 (STEMCELL Technologies) at 37°C, 5% CO_2_ atmosphere, as previously described [62]. Culture medium was replaced every 24 h, and ReLeSR (STEMCELL Technologies) was used for routine passage.

### 4.2 Differentiation of Human Pluripotent Stem Cells Into Liver Organoids

Before initiating differentiation, iPSCs were dissociated into clumps using ReLeSR and plated onto Matrigel coated dishes. When the cells attained 80%–90% confluency, mTESR was replaced with RPMI1640/B27 without insulin (Gibco) and contained 100 ng/mL Activin A (R&D Systems), 20 ng/mL BMP4 (R&D Systems), 8 µmol/L CHIR99021 (Stemgent) and 100 ng/mL FGF2 (R&D Systems) for 4 days. On Day 4, the medium was replaced with RPMI1640/B27 plus supplements (Gibco) containing 10 ng/mL FGF2, 50 ng/mL FGF10 (R&D Systems), 3 µmol/L CHIR99021, 20 ng/mL BMP4 and 10 ng/mL VEGF (STEMCELL Technologies) for 2 days. The cultures were maintained in the same medium for the next two days and additionally supplemented with 2 µmol/L retinoic acid (RA) (Sigma–Aldrich) and 10 ng/mL FGF10. Medium changes were performed every 24 h. For subsequent differentiation, on Day 8, cells were harvested using CTS TrypLE (Gibco) and resuspended in organoid formation medium containing RPMI1640/B27 plus supplement, 50 ng/mL VEGF, 2 µmol/L RA, 20 ng/mL HGF (Peprotech), and 1.5 µmol/L A83‐01 (Sigma–Aldrich). 1 mL/well of this medium was added to wells in 24‐well plates, and polycarbonate isopore membranes (0.1 µm pore size) (EMD Millipore) were floated on the surface to create the ALI. Resuspended cells were then spotted onto the membranes in 1.5 µl droplets, each containing 2 × 10^5^ cells. A total of seven droplets were placed, uniformly spaced, on each membrane per well of a 24‐well plate. The cultures were maintained in the same medium for 4 days, with medium changes every 48 h. Finally, the differentiated cells were cultured in HCM (Lonza) supplemented with 50 ng/mL VEGF, 20 ng/mL HGF, 1.5 µmol/L A83‐01, 20 ng/mL oncostatin M (OSM) (R&D Systems), 0.1 µmol/L dexamethasone (Dex) (Tocris Bioscience) for another 10 days, with medium changes every 48 h. The schematic representation of the differentiation procedure is outlined in Figure 1A.

To compare the ALI‐cultured organoids with matrigel‐embedded organoids, we followed the same differentiation protocol and maintained the same media conditions for both systems. The only step that differed was on day 8, instead of transferring cells on air‐liquid interface, 2 × 10^5^ cells (control‐donor derived) were embedded in a 50 µl droplet of 100% matrigel placed in the center of each well of an ultra‐low attachment 96‐well plate. The matrigel was allowed to polymerize at 37°C for 5 min, after which 200 µl of organoid formation media was gently added to each well without disturbing the droplet. For immunofluorescence analysis, the organoids were isolated from matrigel by pipetting, fixed in 4% paraformaldehyde, and stained according to the whole‐mount staining protocol provided in the Supporting Information file.

### 4.3 Lipid Analysis

Prior to lipid extraction, control (CW10192) and MASH (CW10166) donor‐derived liver organoids were treated with 100 µmol/L oleic acid (OA) (treatment group) or fatty acid‐free BSA (control) for 24 h. For lipid extraction, the organoids were minced using a BioMasher (Polysciences) in cold 50% methanol with 0.1 mol/L HCl. A control without organoids was generated in parallel that served as a ‘no cell’ control for assessing background levels. Lipids were extracted in chloroform using a modified Bligh and Dyer method. Lipids were dried under nitrogen gas and stored at −80°C sealed under nitrogen until analysis. Lipids for the second and third experiments were stored at −80°C for > 1 month. All lipid measurements were performed using liquid chromatography high‐resolution tandem mass spectrometry (LC/MS‐MS) using an orbitrap instrument [71, 72, 73]. Three independent differentiation experiments were performed, with at least two independently formed organoids for each differentiation were assessed in parallel for each experimental condition (with or without OA). The second and third experiments were performed more than 1 year after the first experiment.

The organoid lipidomics presented in this study were compared to the lipids measured by Vvedenskaya et al. from 49 healthy donors and 94 MASH donors and Collin de l'Hortet et al. who examined three healthy and three diseased donors [33, 34]. We filtered the data to only include lipids identified with high confidence in both the published studies and our organoid analysis. To make the studies comparable, we focused on the fold difference of OA‐treated MASH organoids relative to untreated control donor‐derived organoids and the fold difference of MASH liver biopsies relative to healthy donor liver samples.

### 4.4 Organoid Transplantation

All animal experiments were reviewed and approved by Institutional Animal Care and Use Committee (IACUC) of the University of Arizona (#2020‐0677). Male NSG mice (Jackson laboratory; age  =  6 weeks) (*n* = 6) were used for engraftment studies that were housed at the animal facility with a 12‐h light–dark cycle and ad libitum access to water and standard rodent diet. For transplantation, liver organoids were differentiated as previously described here and maintained at the air‐liquid interface. On day 21, the organoids were carefully removed from the polycarbonate filter using tweezers, washed in cold PBS, and then transplanted directly under the kidney capsule of the mice, without embedding them in any matrix. For surgery, mice were anesthetized with isoflurane, and an incision was made in the flank to exteriorize the kidney. A small incision was made in kidney capsule to create a sub‐capsular pocket by gently sliding straight suture‐typing forceps under the kidney capsule and allowing them to open when inside the capsule to create sufficient space for organoid placement. The cut edge of the kidney capsule was lifted with the fine forceps, and control donor‐derived liver organoids (10–12 organoids per mouse) were placed into this pocket and gently pushed under the capsule using blunt forceps to minimize the risk of perforation and vascular injury. Throughout the grafting procedure, hydration of the renal capsule was maintained by periodic application of sterile saline with a cotton‐tipped applicator. When grafting was complete, the kidney was returned to the body cavity. The abdominal wall was closed with sutures, and the skin incision was closed with wound clips. Sham‐operated mice served as controls (*n* = 3). Blood samples were collected for albumin measurement. Four weeks after engraftment, the mice were euthanized and kidney tissue samples were collected for further examination.

### 4.5 Statistical Analysis

Data expressed as mean ± SEM (standard error of the mean) from three independent experiments. Statistical significance was determined using one‐way analysis of variance (ANOVA) with Bonferroni's post hoc test for comparison among three or more groups, and Student's t‐test was used for comparisons between two groups. Statistical analysis was performed using GraphPad Prism 5 (GraphPad Software, San Diego, CA, USA). Differences between groups were considered significant at ∗*p* < 0.05, ∗∗*p* < 0.01, and ∗∗∗*p* < 0.001.

Detailed methods are described in the supporting information file.

## Author Contributions

**Study concept and design**: Minocha E, Gupta AK, and Wertheim JA, **Acquisition of data**: Minocha E, Gupta AK, and Schmidt N, Purdy JG**, Analysis and interpretation of data**: Minocha E, Gupta AK, and Schmidt N, Green RM, Purdy JG, and Wertheim JA, **Drafting of manuscript**: Minocha E, Gupta AK, Schmidt N, Green RM, Purdy JG, and Wertheim JA, **Critical revision**: Minocha E, Gupta AK, Schmidt N, Green RM, Purdy JG, and Wertheim JA.

## Funding

This work was supported in part by the University of Arizona, the National Institute of Diabetes and Digestive and Kidney Diseases (NIDDK) of the National Institutes of Health (NIH) under award number R01DK132873 to JAW, and by the Department of Veterans Affairs, Veterans Health Administration, Office of Research and Development, Biomedical Laboratory Research and Development Merit Review Award I21BX005900 to JAW from the United States Department of Veterans Affairs. The views expressed in this article are those of the authors and do not necessarily reflect the position or policy of the National Institutes of Health, the Department of Veterans Affairs or the United States government.

## Conflicts of Interest

The authors declare no conflicts of interest.

## Supporting information




**Supporting File**: advs76322‐sup‐0001‐SuppMat.docx.

## Data Availability

All mass spectrometry data files have been deposited in Metabolomics Workbench, a NIH Common Fund National Metabolomics Data Repository (NMDR) (www.metabolomicsworkbench.org). The data is accessible using Study ID: ST004708 (DOI: 10.21228/M86Z8F). Metabolomics Workbench/National Metabolomics Data Repository (NMDR) is supported by (grant# U2C‐DK119886), Common Fund Data Ecosystem (CFDE) (grant# 3OT2OD030544) and Metabolomics Consortium Coordinating Center (M3C) (grant# 1U2C‐DK119889). Other datasets that support the findings of this study are available from the corresponding author upon reasonable request.
