# Supplementary material for: MASH Background Confers Enhanced Disease Susceptibility and Acetaminophen Toxicity in iPSC‐Derived Liver Organoids
Source: Adv Sci (Weinh). 2026 Jul 27;13(53):e21373. doi: 10.1002/advs.202521373 (PMC13403214; doi:10.1002/advs.202521373)
Supplement: Supplementary file 1 [file ADVS-13-e21373-s001.docx]

**Supporting Information for:**

**MASH Background Confers Enhanced Disease Susceptibility and Acetaminophen Toxicity in iPSC-Derived Liver Organoids**

**Authors:** Ekta Minocha^1,2,3#^, Ashwani Kumar Gupta^1,2,3#^, Nate Schmidt^2,4^, Richard M Green^5^, John G. Purdy^2,4^, Jason A. Wertheim^1,2,3,6*^

^1^Department of Surgery, University of Arizona College of Medicine, Tucson, AZ, USA.

^2^Bio5 Institute, University of Arizona, Tucson, AZ, USA.

^3^Surgery Service, Southern Arizona VA Health Care System, Tucson, AZ, USA.

^4^Department of Immunobiology, University of Arizona College of Medicine, Tucson, AZ, USA.

^5^Northwestern University Feinberg School of Medicine, Chicago, IL, USA.

^6^Department of Biomedical Engineering, University of Arizona, Tucson, AZ, USA.

^#^Authors contributed equally

* Corresponding author:

Jason A. Wertheim, MD PhD; College of Medicine, University of Arizona, 1501 N. Campbell Ave., P.O. Box 245017, Tucson, AZ 85724, USA. Email: [jwertheim@arizona.edu](mailto:jwertheim@arizona.edu)

**Conflict of Interest**

The authors declare no conflicts of interest.

**Table of Contents:**

Supplementary Methods -----------------------------------------------------------------------------------3

Fig. S1--------------------------------------------------------------------------------------------------------12

Fig. S2--------------------------------------------------------------------------------------------------------13

Fig. S3--------------------------------------------------------------------------------------------------------14

Fig. S4--------------------------------------------------------------------------------------------------------15

Fig. S5--------------------------------------------------------------------------------------------------------16

Fig. S6--------------------------------------------------------------------------------------------------------17

Fig. S7--------------------------------------------------------------------------------------------------------18

Fig. S8--------------------------------------------------------------------------------------------------------19

Fig. S9--------------------------------------------------------------------------------------------------------20

Fig. S10------------------------------------------------------------------------------------------------------21

Fig. S11------------------------------------------------------------------------------------------------------22

Fig. S12------------------------------------------------------------------------------------------------------23

Fig. S13------------------------------------------------------------------------------------------------------24

Table S1------------------------------------------------------------------------------------------------------26

Table S2------------------------------------------------------------------------------------------------------27

Table S3------------------------------------------------------------------------------------------------------28

**Supplementary Methods:**

**Culture of primary human hepatocytes (PHH):** Fresh human hepatocytes were purchased from Yecuris Corporation (20-0002) and cultured on collagen-coated plates in hepatocyte culture media (HCM, Lonza) following manufacturer’s instructions. Culture supernatants were collected after 24 hours of incubation, centrifuged to remove cell-debris, aliquoted and stored at -80°C, and used as positive controls in albumin and urea ELISA assays. For gene expression studies, total RNA was extracted after 24 hours of PHH culture and used as a positive control for the expression of hepatic markers and coagulation factors.

**Live/dead assay:** Organoid viability and cell death were assessed using the LIVE/DEAD Cell Imaging Kit (488/570) (Invitrogen), following the manufacturer’s instructions. Briefly, the organoids were incubated in a live-dead staining solution for 20 minutes at room temperature, after which images were acquired on a Zeiss LSM880 inverted confocal microscope and analyzed using ImageJ software (NIH). The cell-viability was expressed as percentages of live cell area divided by the sum of both live and dead cell area of each organoid.

**RNA isolation and quantitative real-time PCR:** Total RNA was extracted using TRIzol reagent (Invitrogen) and quantified using Nanodrop (Thermo Fisher Scientific). For cDNA synthesis, 2 μg of total RNA was reverse-transcribed using High-Capacity cDNA Reverse Transcription Kit (Applied Biosystems, Foster City, CA, USA) according to manufacturer’s instructions. Quantitative PCR was performed on CFX96 Real-Time PCR Detection System (Bio-Rad Laboratories, Hercules, CA, USA) using SsoAdvanced Universal SYBR Green Supermix (Bio-Rad Laboratories). Fold changes were calculated using 2^-ΔΔCT^ method and values were normalized against the housekeeping gene, TATA-box binding protein (TBP). The primer sequences were obtained from Integrated DNA Technologies and are listed in Table S2.

**Immunostaining:** Monolayer staining: Monolayer cells were fixed with 4% Paraformaldehyde (PFA) (Electron microscopy Sciences) for 10 min at room temperature. After washing with PBS, cells were permeabilized with 0.3% Triton X-100 (Sigma Aldrich) for 10 min at room temperature, followed by blocking with 5% donkey or goat serum in PBS for 1 hour at room temperature. Next, the cells were incubated overnight at 4°C with primary antibodies diluted in 5% donkey serum. Following incubation, cells were washed three times with 1x PBS containing 0.01% Tween-20 (PBST) and subsequently stained with secondary antibodies diluted in 5% donkey serum for 1 hour at room temperature. DAPI (0.01μg/ml) (Thermo Fisher Scientific) was used as a nuclear stain. Cells were washed three times with PBST and imaged under a Zeiss Axio Observer 7 inverted fluorescence microscope (Carl Zeiss AG, Oberkochen, Germany).

Whole-mount staining of liver organoids and vibratome sections of kidneys carrying graft: The organoids were transferred to a “U” bottom 96-well plate and fixed with 4% PFA for 15 minutes at room temperature, followed by permeabilization with 1% Triton X-100 for 10 min at room temperature. After washing with PBS, blocking buffer containing 10% donkey or goat serum in PBS was added for 1 hour at room temperature, followed by overnight incubation at 4°C with primary antibodies diluted in 10% donkey serum. Following incubation, the organoids were washed with PBST for 6 hours at 4°C. Organoids were then incubated with secondary antibodies (diluted in 10% donkey serum) and 0.01μg/ml DAPI at 4°C overnight. Finally, organoids were washed with PBST for 6 hours at 4°C and mounted in VECTASHIELD antifade mounting medium (Vector Laboratories). Fluorescent images were taken using a Zeiss LSM880 inverted confocal microscope.

Kidneys with engrafted organoids were fixed in 4% PFA at 4°C for 1 hour per millimeter thickness of the tissue slab before vibratome sectioning at 100 µm. Kidney sections were then transferred to a flat-bottom 96 well plate and immunostaining was performed using the same method as for the organoids.

Paraffin-embedded organoid sections: The organoids were fixed with 4% PFA for 15 minutes at room temperature, followed by washing with PBS and then embedded in Richard-Allan scientific Histogel (Thermo Fisher Scientific). The histogel block was then dehydrated and embedded in paraffin and sectioned at 5 µm. Paraffin embedded sections were then dewaxed in xylene, rehydrated and subjected to heat-induced epitope retrieval with Tris-EDTA buffer (DAKO). The subsequent staining steps were performed using the same method as for the monolayer staining. The complete list of primary and secondary antibodies used in this study are listed in Table S3.

**Hematoxylin and Eosin (H&E) and picrosirius red staining:** Paraffin embedded organoid sections were dewaxed in xylene, rehydrated and then stained with hematoxylin and eosin staining kit (Vector Laboratories) or picrosirius red stain kit (Abcam) according to manufacturer’s instructions and mounted using DPX mounting medium (Electron Microscopy Sciences). Images were acquired using Leica DMI6000 and LAS X software (Leica) was used for image processing.

**Indocyanine green (ICG) uptake and release assay:** The liver organoids were incubated with 1 mg/ml ICG (Sigma Aldrich) in the medium at 37°C for 4 hours. Following incubation, the medium containing ICG was discarded, and the organoids were washed three times with PBS. The cellular uptake of ICG was examined under the Leica DMI6000 inverted microscope. The organoids were then returned to the fresh culture medium and incubated at 37°C for 19 hours to determine ICG release.

**Albumin production:** The albumin levels in the cell-culture supernatant and blood plasma samples were determined using a human-specific albumin enzyme-linked immunosorbent assay (ELISA) kit (Bethyl, Montgomery TX) following the manufacturer’s protocol.

**CYP3A4 activity and inductivity:** CYP3A4 enzyme activities were assessed by P450-Glo assay (V9001, Promega) in accordance with the manufacturer’s instructions. For CYP3A4 induction assays, organoids were treated with 25 mmol/L rifampicin (Sigma Aldrich) or 0.1% vol/vol DMSO control, dissolved in HCM for 72 hours, with repeated dosing every 24 hours. Quantification of CYP3A4 activity was performed using SpectraMax iD5 multi-mode microplate reader (Molecular Devices).

**Low-density lipoprotein (LDL) uptake cell-based assay:** The liver organoids were incubated with LDL-DyLight 550 (Cayman Chemical) working solution in serum-free HCM for 6 hours at 37°C in 5% CO_2_ atmosphere according to manufacturer’s instructions. Following incubation, the organoids were washed twice with HCM, and LDL uptake was examined under Zeiss LSM880 inverted confocal microscope.

**Periodic Acid Schiff (PAS) staining:** To assess glycogen storage, liver organoids were stained with PAS staining kit (Sigma Aldrich), according to manufacturer’s instructions, and imaged under the Leica DMI6000 inverted microscope.

**Flow cytometry:** Liver organoids were treated with 0.5% Trypsin-EDTA (Thermo Fisher Scientific) at 37°C until the cells began to dissociate into single cells, which were then passed through 70 µm cell-strainers. For intracellular staining, cells were centrifuged and resuspended in fixation buffer containing 2% PFA for 15 minutes at 4°C, followed by permeabilization using 0.05% Triton X-100/PBS for 15 minutes at 4°C and blocking using 1% BSA/PBS blocking solution for 15 minutes. Cells were then incubated with primary antibodies for 30 minutes at 4°C, followed by secondary antibodies for 30 minutes at 4°C in dark. For surface marker staining, cells were directly resuspended in blocking solution followed by incubation with primary and secondary antibodies. Flow cytometry was performed using BD FACS Canto II (BD Biosciences), and data were analyzed using FloJo software (BD Biosciences). The list of primary and secondary antibodies used is provided in Table S3.

**Transmission electron microscopy**: Organoids were fixed in fixative solution containing 4% PFA and 2.5% glutaraldehyde (Electron Microscopy Sciences) in 0.1M phosphate buffer (pH 7.4), overnight at 4°C. Post-fixation sectioning and imaging were all performed at the Electron Microscopy Imaging Core facility at the University of Arizona, according to the standard protocol. 70 nm ultrathin sections of each organoid were obtained using a Leica UltraCut UC6 ultramicrotome. Sections were mounted on 200 mesh copper grids and images were acquired on a FEI Tecnai G2 Spirit BT TEM.

**Visualization of bile canaliculi formation:** Liver organoids were incubated in HCM containing 2 µmol/L 5-(and-6) carboxy-2’,7’-dichlorofluoroscein diacetate (CDFDA) and 1 µg/ml Hoechst 33342 (Invitrogen) for 30 min at 37°C. Subsequently, the organoids were washed twice with HCM and observed under a Zeiss LSM880 inverted confocal microscope.

**Rhodamine123 transport assay:** To determine Multi Drug Resistance Protein 1 (MDR1) activity, organoids were incubated with 100 µmol/L Rhodamine123 (Sigma Aldrich), for 5 minutes at 37°C. The samples were washed three times to remove rhodamine 123, and incubated with culture medium for another 40 minutes at 37°C. To demonstrate that Rhodamine123 transfer was indeed mediated by MDR1, organoids were incubated with 10 µmol/L verapamil (Sigma Aldrich), an MDR1 inhibitor, for 30 minutes at 37°C, and the rhodamine assay was repeated. Following completion of each experiment, images were acquired using a Zeiss LSM880 inverted confocal microscope and analyzed using ImageJ software (NIH).

**Live perfusion of organoids with Ulex Europaeus Agglutinin l (UEA-1):** UEA-1 is a lectin that binds to glycoproteins containing alpha-linked fucose residues on the surface of endothelial cells in human tissues [1]. Organoids were treated with 5 µg/ml UEA-1 DyLight 594 (Vector Laboratories) in HCM for 1 hour at 37°C. Following incubation, organoids were washed three times with PBS, fixed using 4% PFA for 15 minutes at room temperature and then whole-mount immunofluorescence staining for CD31 was performed as described earlier. Fluorescence images were acquired using a Zeiss LSM880 inverted confocal microscope.

**Lipopolysaccharide (LPS) treatment:** Liver organoids were exposed to LPS treatment in HCM media for 24 hours at 0, 200 ng/ml and 1µg/ml concentration (0 ng/ml LPS represents untreated control) in 96-well ultra-low attachment multi-well plates (1 organoid/well with 100ul treatment media) at 37°C. Following treatment, organoids were washed twice in PBS and further assayed for analysis of inflammatory genes. The supernatants were collected and stored at -80^ο^C to evaluate the concentrations of secreted cytokines: human IL-6 and IL-8 using commercial ELISA kits from R&D Systems, following the manufacturer’s protocol. For each cell line, supernatants from six wells of a 96-well plate were pooled per condition (0, 200 ng/ml and 1µg/ml LPS), and samples were processed in duplicate.

**Free-fatty acid (FFA) exposure:** Liver organoids were exposed to 100 µmol/L oleic acid (Sigma-Aldrich) in HCM for 24 hours at 37°C in 96-well ultra-low attachment multi-well plates (Corning), and organoids treated with fatty-acid free BSA served as controls. For visualization of lipid droplets, organoids were washed with PBS and incubated with 2 μmol/L BODIPY 493/503 (ThermoFisher Scientific) staining solution for 30 minutes in the dark at 37°C. Hoechst 33342 was used to stain the nuclei and images were taken on Zeiss Axio Observer 7 (Carl Zeiss) inverted fluorescence microscope. The supernatant was assayed for human ACTA2 (Abcam), TGF-β1, IL-8 and IL-23 (R&D Systems) by ELISA, according to the manufacturer’s instructions. Presence of pro-collagen type 1 was detected using the Pro-collagen Type 1C-peptide ELISA kit (Takara Bio Inc.). For each cell line, supernatants from eight wells of a 96-well plate were pooled per condition (NTC and OA treated), and samples were processed in duplicate for the analysis of TGF-β1, IL-8, IL-23 and pro-collagen type 1. Additionally, cell-lysates from eight organoids per condition (NTC and OA treated) were pooled and processed in duplicate for the detection of ACTA2.

**Drug treatment and cell-toxicity tests:** Liver organoids were exposed to acetaminophen (APAP) (Sigma Aldrich) treatment in HCM media for 72 hours at 0, 5, 25 and 50 mmol/L concentration (0 mmol/L APAP represents untreated control) in 96-well ultra-low attachment multi-well plates. Following treatment with APAP, organoids were washed twice in PBS and then stained with fluorescent probes: mBCL (5 x 10^-6^ mol/L) and DHE (10 x 10^-6^ mol/L) (both from Sigma Aldrich) to determine the levels of glutathione (GSH) and reactive oxygen species (ROS), respectively. The organoids were incubated with probes for 20 min at 37°C, and signals from each probe were detected using a Zeiss LSM880 inverted confocal microscope. Transferase-Mediated dUTP Nick End-Labeling (TUNEL) assay was performed to determine the APAP-induced apoptotic cell-death using ApopTag Plus In-Situ Apoptosis Fluorescein Detection Kit (Millipore) following the manufacturer’s instruction.

**Mitochondrial ROS detection:** Mitochondrial superoxide levels were detected using the fluorescent dye MitoSOX Red (Invitrogen) after treating liver organoids with APAP or OA+ APAP. For staining, organoids were washed with PBS and incubated in medium containing 5µM MitoSOX Red and 10 µg/ml Hoechst 33342 for 30 minutes at 37^ο^C in a 5% CO_2_ incubator in the dark. After washing with PBS, images were acquired using a Leica DMI6000 microscope and analyzed using ImageJ software (NIH).

**Mitochondrial Membrane Potential (MMP) assessment:** To determine changes in the MMP following APAP and OA+APAP treatment, liver organoids were incubated with 15 µM JC-1 (Invitrogen) staining solution in HCM culture media for 30 minutes at 37^ο^C, 5% CO_2_ in the dark. After incubation, the organoids were washed with PBS, and images were acquired using a Leica DMI6000 microscope and analyzed using ImageJ software (NIH)

**ATP measurement:** ATP levels were measured using an ATP assay kit (Abcam) according to the manufacturer’s protocol. Following treatment with APAP and OA+APAP, both control and MASH donor derived liver organoids were homogenized in ATP assay buffer, and proteins were removed using the Deproteinizing Sample Preparation Kit- TCA (Abcam, ab204708). The fluorometric detection was performed at excitation/emission wavelengths of 535/587 nm using CLARIOstar Plus microplate reader (BMG Labtech) and results were expressed as relative fluorescence units (RFU).

**Lipid analysis:** Prior to lipid extraction, control (CW10192) and MASH (CW10166) donor-derived liver organoids were treated with 100 µmol/L oleic acid (treatment group) or fatty-acid free BSA (control) for 24 hours. For lipid extraction, the organoids were minced using a BioMasher (Polysciences) in cold 50% methanol with 0.1 mol/L HCl. A control without organoids was generated in parallel that served as a ‘no cell’ control for assessing background levels. Lipids were extracted in chloroform using a modified Bligh and Dyer method. Extracted lipids were dried under nitrogen gas and stored at -80° C sealed under nitrogen until analysis. Lipids for the second and third experiments were stored at -80° C for > 1 month. All lipid measurements were performed using liquid chromatography high-resolution tandem mass spectrometry (LC/MS-MS) using an orbitrap instrument [2–4]. A solution of 1:1:1 MeOH:Isopropanol:Chloroform was used to resuspend lipids, that were separated by reverse-phase chromatography on a Vanquish ultra high-performance LC (UHPLC) system on a Kinetex C18 reverse-phase column (Phenomenex) at 60°C using two solvents: solvent A (40:60 water:MeOH with 10 mM ammonium formate and 0.1% formic acid) and solvent B (10:90 MeOH:Isopropanol with 10 mM ammonium formate and 0.1% formic acid). UHPLC was performed at a flow rate of 0.25 mL/min for 30 minutes. The gradient was established at 25% solvent B for 2 minutes, increased to 65% solvent B from 2 minutes to 4 minutes, held at 65% solvent B from 4 minutes to 5 minutes, increased to 100% solvent B from 5 minutes to 16 minutes, held at 100% solvent B from 16 minutes to 22 minutes, decreased to 25 % solvent B from 2 minutes to 23.5 minutes. The column was briefly washed and equilibrated after each run. Blank samples were run before and after samples. The autosampler temperature was 7°C. Lipids were measured using a Thermo Scientific Orbitrap Exploris 240 operating in a full MS1/data dependent MS2 (dd-MS2) TopN mode with 5 dd-MS2 scans. Lipids were ionized using heated electrospray ionization. Each sample was analyzed in positive and negative ion mode. For positive mode, 8 µl of samples were analyzed using a sheath gas of 45, auxiliary gas of 25, sweep gas of 1, spray voltage of 4 kV, RF lens of 110%, ion transfer tube temperature of 320°C, and a vaporization temperature of 220°C. For negative mode, 10 µl of sample was analyzed using a sheath gas of 20, auxiliary gas of 10, sweep gas of 1, spray voltage of 3.8 kV, RF lens of 110%, ion transfer tube temperature of 260°C and vaporization temperature of 170°C. MS1 was collected at a 90,000 resolution with a transient time of 192 ms, normalized automatic gain control (AGC) was set to standard and maximum injection time was set to auto. MS1 spectra were collected from 200-2000 m/z. MS2 spectra were collected at 30,000 resolution, normalized AGC of 100%, and maximum injection time of 64 ms. Normalized collision energy value 23 was used in both positive and negative modes. The instrument was calibrated weekly, and the internal calibration source “Easy-IC” was used in Scan-to-Scan mode.

Lipids were identified using Maven 2 software and quantitatively measured using the AreaTop feature [5]. Lipid identification was confirmed using the following MS2 data: TG through neutral loss of fatty acyl tail (positive mode; [M+NH4]+), DG through neutral loss of fatty acyl tail (positive mode; [M+NH4]+), CE fragment of 369.351 (positive mode; [M+NH4]+), PC fragment of 184.074 (positive mode), PE fragment of 196.038 or the tail plus 196.038 (negative mode) and loss of 141.019 (positive mode), PG fragment of 152.996 plus the identification of the FA tails (negative mode), PI fragment of 241.012 (negative), and PS neutral loss of 87.032 (negative). PC lipids were further confirmed via M+HCOO- identification in negative mode. Both PC and PS tails were identified in negative mode.

**Lipid comparison:** The organoid lipidomics presented in this study was compared to the lipids measured by Vvedenskaya et al. from 49 healthy donors and 94 MASH donors and Collin de l’Hortet et al. who examined 3 healthy and 3 diseased donors [6,7]. Data from Collin de l’Hortet et al. was obtained from Metabolomics Workbench Study ID: ST001185. Peak area was used to determine the fold change of MASH donors relative to healthy control. Data from Vvedenskaya et al. were obtained from supplemental materials of the publication. The normalized lipid values were used to determine the fold change of MASH donors relative to healthy donors that lacked liver disease and were non-obese. In both studies, disease was defined according to MASLD activity score and body-mass index. We filtered the data to only include lipids identified with high confidence in both the published studies and our organoid analysis. To make the studies comparable, we focused on the fold difference of OA-treated MASH organoids relative to untreated control donor-derived organoids and the fold difference of MASH liver biopsies relative to healthy donor liver samples.

In positive mode, PC [M+H]+, CE [M+NH4]+, DG [M+NH4]+, and TG [M+NH4]+) were included. In negative mode, PA [M-H]-, PE [M-H]-, PG [M-H]-, PI [M-H]-, PS [M-H]-, and PC [M+HCOO]- were included. Low quality identifications were removed, including those with grade D from LipidSearch. Only, even chain lipids were included. Molecularly identified lipids were pooled to generate a sum composition of lipid species (i.e., TG(16:0_14:1_18:1,) and TG(16:1_14:0_18:1) became TG(48:2)). No imputation was performed: lipids had to be present in all organoid samples, as well as in six samples from Collin de l'Hortet et al., and 25 (healthy control) and 48 (MASH) from Vvedenskaya et al. Statistical testing was performed in Excel and RStudio. Heatmaps and Venn diagrams were designed using RStudio software.

**Supplementary Figures:**

**Fig. S1.** **Characterization of endoderm and mesoderm co-differentiation from human iPSCs:** (A) Representative immunofluorescence images of control (C1, C2) and MASH (M1, M2) donor iPSCs (day 0) depict the expression of pluripotency markers (OCT4 and TRA-1-60). (B) Representative immunofluorescence images of control and MASH donor iPSCs on day 4 show the expression of endodermal (GATA4, FOXA2) and mesodermal (TBXT) markers, with very few cells expressing ectodermal marker (MAP2) (scale bar: 100µm).

**Fig. S2.** **Live/dead assay on liver organoids:** (A) Representative whole-mount immunofluorescence images of live/dead staining on control (C1, C2) and MASH (M1, M2) donor-derived liver organoids on day 21 (scale bar: 500µm). (B) Graph shows quantification of cell-viability via ImageJ, expressed as percentage values. Data presented as mean ± SEM. (n=3) n.s. non-significant (one-way ANOVA).

**Fig. S3. Liver organoids exhibit an extensive, interconnected vascular network:** (A) Image interpretation of immunoﬂuorescence staining for LYVE1 in control (C1, C2) and MASH (M1, M2) donor-derived liver organoids on day 21, generated by AngioTool analysis, depict vast, interconnected vascular networks (scale bar: 500µm) (B) AngioTool analysis of % vessel area, number of vascular junctions, and average vessel length based on LYVE1 immunoﬂuorescence staining of liver organoids. Data presented as mean ± SEM. (n=4, n.s. non-significant).

**Fig. S4. Liver organoids exhibit epithelial as well as supportive cell lineages:** Representative flow cytometric histograms show the expression of ALB, CK19, CD166/ALCAM, LYVE1, and CD68 in control and MASH donor-derived liver organoids. Histogram analysis was performed using FlowJo software.

**Fig. S5. Cellular characterization of liver organoids:** (A) Representative phase-contrast images of whole-mount control (C1, C2) and MASH (M1, M2) donor-derived liver organoids on day 21, scale bar: 500µm. (B) Representative immunofluorescent images of control and MASH donor-derived liver organoids on day 21 depict the hepatocyte marker (ALB) and endothelial markers (LYVE1, CD31) in wholemount organoids, along with histological sections showing cholangiocyte marker (SOX9), stellate cell marker (ALCAM), and Kupffer cell marker (CD68, scale bar: 500µm).

**Fig. S6.** **Cellular characterization of liver organoids cultured under air-liquid interface (ALI) or Matrigel-embedded conditions:** (A) Phase-contrast image of liver organoids on day 21 of differentiation cultured under matrigel-embedded conditions (scale bar: 250µm) (B) Gene-expression analysis of lineage-specific markers on day 21 of differentiation in cultures maintained under ALI or within matrigel-embedded conditions. Values determined relative to *TBP* and presented as fold change relative to expression in ALI culture conditions, which is set as 1 (n=3). (C) Representative whole-mount immunofluorescence images of control donor-derived liver organoids showing LYVE1+ and CD31+ endothelial networks in organoids cultured under ALI and matrigel-embedded conditions. Both images were taken at same magnification (scale bar: 250µm) (D) AngioTool analysis of the corresponding LYVE1 immunofluorescence projections, where thick red lines represent vascular paths and blue dots represent vascular junction points. (E) Quantification of LYVE1+ vasculature using AngioTool shows the percent vessel area, number of vascular junctions, and average vessel length (n=3). Data presented as mean ± SEM. ∗p < 0.05, ∗∗p < 0.01, ∗∗∗p < 0.001, n.s. non-significant (Student’s t-test).

**Fig. S7.** **Liver organoids express transcripts for coagulation factors:** (A) Gene-expression analysis of coagulation factors F2, F5, F7, F8, and F10 in control and MASH donor-derived liver organoids at day 21 of differentiation. Values determined relative to *TBP* and presented as fold change relative to expression in day 0 iPSC, which is set to 1. (n=4). Data presented as mean ± SEM. ∗p < 0.05, ∗∗p < 0.01, ∗∗∗p < 0.001 (one-way ANOVA). (B). Coagulation factor gene-expression is shown relative to primary human hepatocytes (PHH), used as positive controls and set to 1 (n=4). Data presented as mean ± SEM.

**Fig. S8.** **Liver organoids secrete inflammatory cytokines in response to LPS treatment**: Quantification by ELISA of secreted IL-6 and IL-8 levels in cell-culture supernatants from MASH and control liver organoids following LPS treatment (n=4). Data presented as mean ± SEM. ∗p < 0.05, ∗∗p < 0.01, ∗∗∗p < 0.001 (one-way ANOVA).

**Fig. S9. Lipid accumulation in iPSC-derived organoids from normal and MASH donors:** (A) Representative whole-mount BODIPY staining images show the accumulation of neutral lipid droplets in BSA (vehicle-control) and 25, 50 and 100 µM oleic acid (OA) treated MASH and control-donor liver organoids (scale bar: 500µm). (B) Quantification of BODIPY fluorescence intensity using ImageJ (n=4). Data presented as mean ± SEM. (n=4). ∗p < 0.05, ∗∗∗p < 0.001 (one-way ANOVA).

**Fig. S10. Modelling steatohepatitis pathology using liver organoids:** (A) Representative whole-mount live imaging of BODIPY staining shows the accumulation of neutral lipid droplets in oleic acid (OA) and BSA (vehicle-control) treated control and MASH donor-derived liver organoids. Graph shows ImageJ quantification of BODIPY fluorescence intensity (n=4). (B) Representative immunofluorescent images of histological sections from control and MASH donor-derived liver organoids show the expression of fibrosis markers: COL1A1 and ACTA2 in response to OA treatment (scale bar: 500µm). Graphs show ImageJ quantification of % positive area staining for COL1A1 and ACTA2 (n=4). Data presented as mean ± SEM. (n=4). ∗p < 0.05, ∗∗p < 0.01, and ∗∗∗p < 0.001 (one-way ANOVA).

**Fig. S11. Lipid profiles of MASH organoids are comparable to those observed in human MASH liver biopsies:** Lipids significantly upregulated (A, C) or downregulated (B, D) in organoids were compared to those increased or decreased in human liver biopsies of MASH relative to healthy human liver. Lipids were transformed and filtered to be equivalent in all three studies as described in the Experimental section. (A, C) Lipid levels were considered upregulated when fold change > 1.5 and p < 0.05. (B, D) Lipid levels were considered downregulated when fold change < 0.67 and p <0.05. One-sample T-tests were performed on normalized data of our data set and Collin de l’Hortet et al., and Mann-Whitney U test was performed on normalized data of Vvedenskaya et al. Our data set, n=6; Collin de l’Hortet, n=3; Vvedenskaya et al., n=49 (healthy control), n=94 (MASH Liver).

**Fig. S12. Modelling drug-induced cytotoxicity using liver organoids.** (A) Representative fluorescence images show apoptosis using TUNEL staining in whole-mount control and MASH donor liver organoids treated with 5, 25 and 50 mM APAP (scale bar: 500µm). (B) Graph depicts ImageJ quantification of apoptosis represented as TUNEL^+^ cells (%). Data presented as mean ± SEM. (n=4). ∗∗∗p < 0.001 (one-way ANOVA).

**Fig. S13. APAP exposure leads to mitochondrial oxidative stress and mitochondrial dysfunction:** (A) Representative whole-mount fluorescence images of control and MASH-donor derived liver organoids show MitoSOX Red staining to assess mitochondrial superoxide levels following treatment with 25 mM APAP alone or with 100µM OA (scale bar: 500µm). (B) Representative whole-mount fluorescence images of control and MASH-donor derived liver organoids show changes in mitochondrial membrane potential (MMP) detected by JC-1 staining (scale bar: 500µm). Red fluorescence represents JC-1 aggregates, indicating energetic mitochondria with intact MMP, while green fluorescence represents JC-1 monomers, indicating decreased MMP with less energetic mitochondria. Graphs depict ImageJ quantification of (C) relative levels of mitochondrial ROS (mtROS) detected by MitoSOX Red (n=4), and (D) ratiometric analysis of JC-1 red and green fluorescence intensity (n=4). (E) Quantification of cellular adenosine triphosphate (ATP) levels. Values presented as relative fluorescence unit (RFU) normalized to protein concentration (n=4). Data presented as mean ± SEM. ∗p < 0.05, ∗∗p < 0.01, ∗∗∗p < 0.001 (one-way ANOVA).

**Table S1:** Comparison of air-liquid interface (ALI) versus Matrigel-embedded organoid culture

| **Category** | **Matrigel-embedded liver organoids** | **Air-liquid interface (ALI)-based liver organoids** |
| --- | --- | --- |
| **Differentiation Efficiency** | Express lower levels of   - hepatocyte marker (ALB), - cholangiocyte marker (CFTR and SLC10A2), and - sinusoidal endothelial-cell specific markers (LYVE1 and CD31), compared to ALI   Express higher levels of   - Stellate-cell marker (DES) - Kupffer-cell marker (CD68), compared to ALI - Immunofluorescence confirms lower levels of LYVE1+ and CD31+ endothelial networks, compared to ALI | Express higher levels of   - hepatocyte marker (ALB), - cholangiocyte marker (CFTR and SLC10A2), and - sinusoidal endothelial-cell specific markers (LYVE1 and CD31), compared to Matrigel-embedded organoids.   Express lower levels of   - Stellate-cell marker (DES) - Kupffer-cell marker (CD68), compared to Matrigel-embedded organoids. - Immunofluorescence confirms presence of vast endothelial networks positive for LYVE1 and CD31 in ALI organoids |
| **Operational Simplicity** | - Requires strict temperature control due to Matrigel’s thermal sensitivity - Organoid retrieval from Matrigel necessitates mechanical dissociation, introducing additional cellular stress | - All procedures can be performed at room temperature. - Organoids can be collected without mechanical dissociation |
| **Cost** | - Additional cost due to Matrigel use | - No Matrigel-related cost |
| **Reproducibility** | - Organoid size and cell number per organoid vary due to self-organization of cells within Matrigel droplets | - Organoid size and cell-number per organoid are more consistently controllable in ALI culture |

**Table S2:** List of qPCR primer sequences

| **Gene** | **Forward primer** | **Reverse primer** |
| --- | --- | --- |
| OCT4 | CCTGAAGCAGAAGAGGATCACC | AAAGCGGCAGATGGTCGTTTGG |
| NANOG | CTCCAACATCCTGAACCTCAGC | CGTCACACCATTGCTATTCTTCG |
| SOX1 | GAGTGGAAGGTCATGTCCGAGG | CCTTCTTGAGCAGCGTCTTGGT |
| PAX6 | CTGAGGAATCAGAGAAGACAGGC | ATGGAGCCAGATGTGAAGGAGG |
| MIXL1 | GGCGTCAGAGTGGGAAATCC | GGCAGGCAGTTCACATCTACC |
| EOMES | AAATGGGTGACCTGTGGCAAAGC | CTCCTGTCTCATCCAGTGGGAA |
| GATA4 | CGACACCCCAATCTCGATATG | GTTGCACAGATAGTGACCCGT |
| SOX17 | GTGGACCGCACGGAATTTG | GGAGATTCACACCGGAGTCA |
| FOXA2 | GGAGCAGCTACTATGCAGAGC | CGTGTTCATGCCGTTCATCC |
| ALB | TGCAACTCTTCGTGAAACCTATG | ACATCAACCTCTGGTCTCACC |
| SERPINA1 | ATGCTGCCCAGAAGACAGATA | CTGAAGGCGAACTCAGCCA |
| HNF4A | CGAAGGTCAAGCTATGAGGACA | ATCTGCGATGCTGGCAATCT |
| CYP3A4 | GTGGGGCTTTTATGATGGTCA | GCCTCAGATTTCTCACCAACACA |
| KRT7 | CATCGAGATCGCCACCTACC | GATATTCACGGCTCCCACTCC |
| CFTR | GGAGAGCATACCAGCAGTGACT | TTCCAAGGAGCCACAGCACAAC |
| SLC10A2 | GGACAATGCAACAGTTTGCTC | CCGTACTTAGGACCACACTTAGG |
| AQP1 | TATGCGTGCTGGCTACTACCGA | GGTTAATCCCACAGCCAGTGTAG |
| PECAM1 | AAGTGGAGTCCAGCCGCATATC | ATGGAGCAGGACAGGTTCAGTC |
| LYVE1 | AGGCTCTTTGCGTGCAGAA | GGTTCGCCTTTTTGCTCACAA |
| CD68 | CGAGCATCATTCTTTCACCAGCT | ATGAGAGGCAGCAAGATGGACC |
| ALCAM | TCCAGAACACGATGAGGCAGAC | GTAGACGACACCAGCAACAAGG |
| DESMIN | TCCAGTCCTACACCTGCGAGAT | CGCAATGTTGTCCTGGTAGCCA |
| TNF-α | GAGGCCAAGCCCTGGTATG | CGGGCCGATTGATCTCAGC |
| IL-6 | CCTGAACCTTCCAAAGATGGC | TTCACCAGGCAAGTCTCCTCA |
| TGF-β | CAATTCCTGGCGATACCTCAG | GCACAACTCCGGTGACATCAA |
| COL1A1 | GAGGGCCAAGACGAAGACATC | CAGATCACGTCATCGCACAAC |
| IL-23 | GAGCCTTCTCTGCTCCCTGATA | GACTGAGGCTTGGAATCTGCTG |
| GCLC | GGAAGTGGATGTGGACACCAGA | GCTTGTAGTCAGGATGGTTTGCG |
| GCLM | TCTTGCCTCCTGCTGTGTGATG | TTGGAAACTTGCTTCAGAAAGCAG |
| GPX1 | GTGCTCGGCTTCCCGTGCAAC | CTCGAAGAGCATGAAGTTGGGC |
| NQO1 | CCTGCCATTCTGAAAGGCTGGT | GTGGTGATGGAAAGCACTGCCT |
| F2 | ATGGGCTGGATGAGGACTCAGA | CGGTTTTGTCCTCCAGCGACTT |
| F5 | GCCAGACCTTGCTGGAAAATGG | CCAACCTCTGTGTTTAGGAGCC |
| F7 | CCTCAAGTCCATGCCAGAATGG | CACAGATCAGCTGGTCATCCTTG |
| F8 | CCAGAGTTCCAAGCCTCCAACA | GGAAGTCAGTCTGTGCTCCAATG |
| F10 | TGGTGGAACCATTCTGAGCGAG | CGGTTGTGCTTGATGACCACCT |
| TBP | TGTATCCACAGTGAATCTTGGTTG | GGTTCGTGGCTCTCTTATCCTC |

**Table S3**: List of antibodies/lectins used in this study

| **Antibodies** | **Host** | **Supplier** | **Catalog number** | **Dilution** |
| --- | --- | --- | --- | --- |
| OCT4 | Rabbit | Invitrogen | A13998 | 1:100 |
| TRA-1-60 | Mouse | Invitrogen | MA1-023 | 1:100 |
| GATA4 | Rabbit | Cell signaling Tech | 36966S | 1:200 |
| FOXA2 | Mouse | Abcam | ab60721 | 1:100 |
| MAP2 | Rabbit | Abcam | ab32454 | 1:100 |
| TBXT | Goat | R&D | AF2085 | 1:100 |
| ALB | Chicken | Abcam | ab106582 | 1:100 |
| ALB | Goat | Bethyl Laboratories | A80-129A | 1:100 |
| ALB | Mouse | R&D | IC1455A | 1:100 |
| LYVE1 | Rabbit | Abcam | ab219556 | 1:100 |
| PECAM1 | Mouse | Cell signaling Tech | 3528 | 1:50 |
| PECAM1 | Rat | BD Pharmingen | 550274 | 1:50 |
| SOX9 | Rabbit | Abcam | ab185230 | 1:100 |
| CD68 | Rabbit | Abcam | ab213363 | 1:100 |
| ALCAM | Rabbit | Abcam | ab109215 | 1:100 |
| ALCAM | Mouse | Biolegend | 343903 | 1:100 |
| CFTR | Mouse | Millipore | MAB3484 | 1:100 |
| CK19 | Mouse | Abcam | ab7754 | 1:100 |
| CK19 | Mouse | Invitrogen | MA5-28576 | 1:100 |
| MRP2 | Mouse | Millipore | MAB4150 | 1:100 |
| A1AT/SEPINA1 | Rabbit | Agilent DAKO | A0012 | 1:100 |
| HNF4A | Rabbit | Cell signaling Tech | 3113 | 1:100 |
| ZO-1 | Mouse | Invitrogen | 33-9100 | 1:100 |
| DESMIN | Mouse | BD | 550626 | 1:100 |
| Ac-αTubulin | Mouse | Proteintech | 66200-1-Ig | 1:100 |
| COL-1 | Mouse | Abcam | ab6308 | 1:100 |
| α-SMA | Rabbit | Abcam | Ab124964 | 1:100 |
| HuNu | Mouse | Millipore Sigma | MAB1281B | 1:50 |
| NuMa | Rabbit | Abcam | ab97585 | 1:100 |
| RBC | Rabbit | Rockland | 110-4139 | 1:100 |
| UEA1 | - | Vector Lab. | DL-1067-1 | 1:200 |
| Donkey anti-Mouse IgG Alexa Flour 488 | Donkey | Invitrogen | A21202 | 1:200 |
| Donkey anti-Mouse IgG Alexa Flour 568 | Donkey | Invitrogen | A10037 | 1:200 |
| Goat anti-Chicken IgG Alexa Flour 488 | Goat | abcam | ab150169 | 1:200 |
| Donkey anti-Rabbit IgG Alexa Flour 488 | Donkey | Invitrogen | A21206 | 1:200 |
| Donkey anti-Rabbit IgG Alexa Flour 568 | Donkey | Invitrogen | A10042 | 1:200 |
| Donkey anti-Goat IgG Alexa Flour 568 | Donkey | Invitrogen | A11057 | 1:200 |

**References:**

[1]	Hormia M, Lehto V-P, Virtanen I. Identification of UEA I-binding surface glycoproteins of cultured human endothelial cells. Cell Biol Int Rep 1983;7:467–75. https://doi.org/https://doi.org/10.1016/0309-1651(83)90136-4.

[2]	Yuecheng X, Lena L, Ian K, et al. Human Cytomegalovirus Uses a Host Stress Response To Balance the Elongation of Saturated/Monounsaturated and Polyunsaturated Very-Long-Chain Fatty Acids. MBio 2021;12:10.1128/mbio.00167-21. https://doi.org/10.1128/mbio.00167-21.

[3]	Yuecheng X, Samuel H, M WL, et al. Human Cytomegalovirus pUL37x1 Is Important for Remodeling of Host Lipid Metabolism. J Virol 2019;93:10.1128/jvi.00843-19. https://doi.org/10.1128/jvi.00843-19.

[4]	Ganji R, Paulo JA, Xi Y, et al. The p97-UBXD8 complex regulates ER-Mitochondria contact sites by altering membrane lipid saturation and composition. Nat Commun 2023;14:638. https://doi.org/10.1038/s41467-023-36298-2.

[5]	Seitzer P, Bennett B, Melamud E. MAVEN2: An Updated Open-Source Mass Spectrometry Exploration Platform. Metabolites 2022;12:684. https://doi.org/10.3390/metabo12080684.

[6]	Vvedenskaya O, Rose TD, Knittelfelder O, et al. Nonalcoholic fatty liver disease stratification by liver lipidomics. J Lipid Res 2021;62. https://doi.org/10.1016/j.jlr.2021.100104.

[7]	Collin de l’Hortet A, Takeishi K, Guzman-Lepe J, et al. Generation of Human Fatty Livers Using Custom-Engineered Induced Pluripotent Stem Cells with Modifiable SIRT1 Metabolism. Cell Metab 2019;30:385-401.e9. https://doi.org/10.1016/j.cmet.2019.06.017.
